# Supplementary material for: IP3 Receptors Preferentially Associate with ER-Lysosome Contact Sites and Selectively Deliver Ca2+ to Lysosomes
Source: Cell Rep. 2018 Dec 11;25(11):3180–3193.e7. doi: 10.1016/j.celrep.2018.11.064 (PMC6302550; doi:10.1016/j.celrep.2018.11.064)
Supplement: Document S1. Figures S1–S7 and Table S1 [file mmc1.pdf]

Cell Reports, Volume *25*

Supplemental Information

IP_3 Receptors Preferentially Associate with ER-Lysosome Contact Sites and Selectively Deliver Ca^{2+} to Lysosomes

Peace Atakpa, Nagendra Babu Thillaiappan, Stefania Mataragka, David L. Prole, and Colin W. Taylor

Table S1 Primers Used for Constructing and Sequencing Plasmids. Related to STAR methods.

Primer name	Sequence
GGECO1.2F	CATGGATCCATGGTCGACTCATCACGTCGTAAG
GGECO1.2R	GTAGAATTCCTACTTCGCTGTCATCATTTG TACAAACTCTTC
LAMP1F	TACAAGCTTGCTTCGAATTCTCGCCACCAT
LAMP1R	GGTGGATCCTCCTGAACCTCCGATGGTCTG ATAGCCCGCG
LAMP1SeqF1	ACGTTCAGCACCTCCAACTA
LAMP1SeqF2	ATCGGCAGGAAGAGGAGTCA
T7 promoter (F)	TAATACGACTCACTATAGGG
GGECO1.2SeqM	CAAACCCCAGTGTGTCCAAG
GGECO1.2SeqE	GCCTACCACTACCAGCAGAA

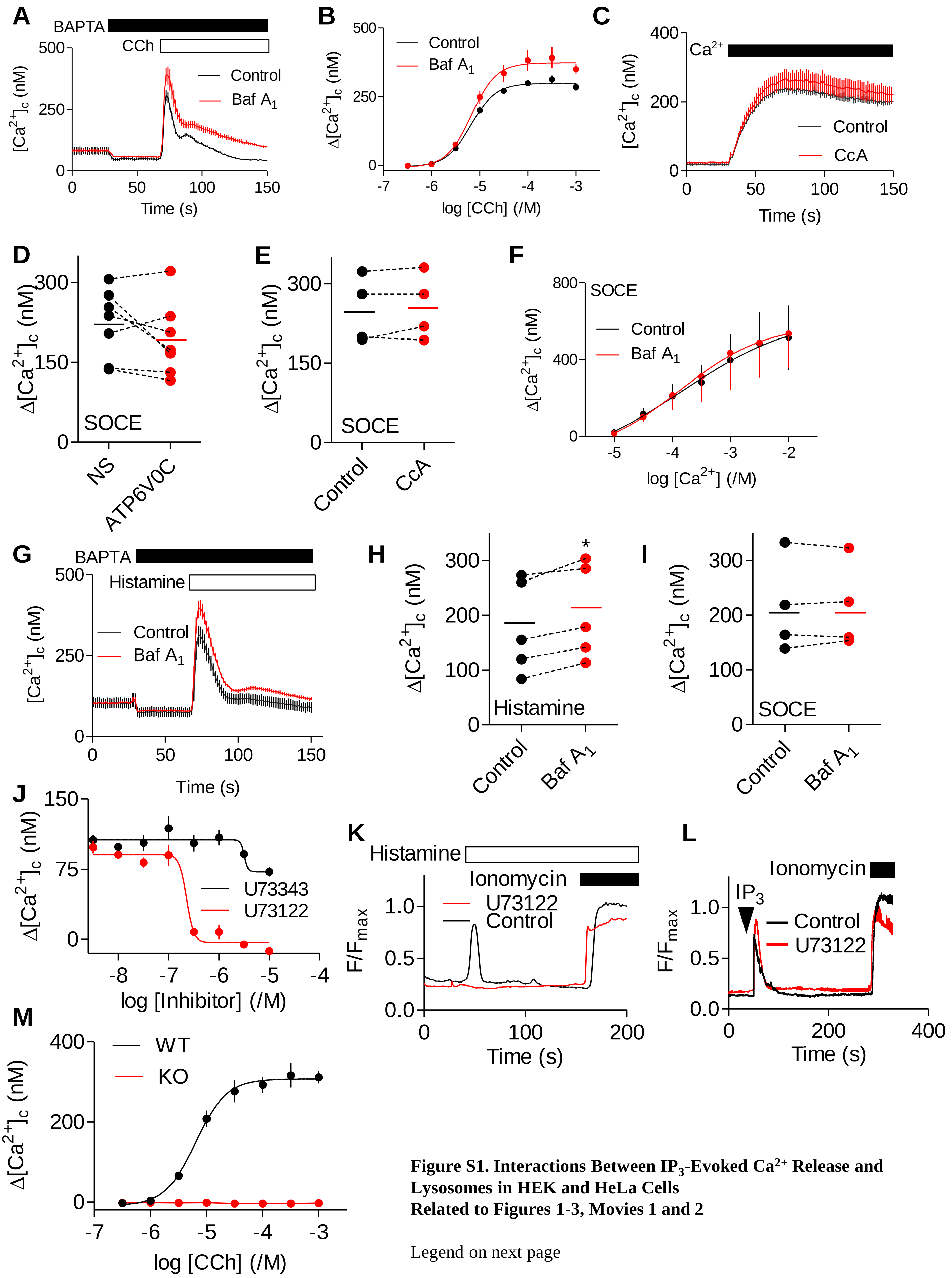

Figure S1. Interactions Between IP_3-Evoked Ca^{2+} Release and Lysosomes in HEK and HeLa Cells
Related to Figures 1-3, Movies 1 and 2

Legend on next page

Figure S1. Interactions Between IP_3-Evoked Ca^{2+} Release and Lysosomes in HEK and HeLa Cells

Figure on preceding page

(A) Fluo 8-loaded HEK cells were treated with bafilomycin A_1 (Baf A_1, 1 μM, 1 hr) in HBS before addition of BAPTA (2.5 mM) to chelate extracellular Ca^{2+} and then CCh (1 mM) to stimulate IP_3 formation. Typical results show means ± SD from 3 wells in a single experiment.
(B) Summary results (mean ± SEM, n = 5) show effects of CCh on Ca^{2+} release with and without Baf A_1.
(C) HEK cells were treated with thapsigargin (1 μM, 15 min) in Ca^{2+}-free HBS to activate SOCE, before restoration of extracellular Ca^{2+} (10 mM) alone or after treatment with CcA (1 μM, 1 hr). Typical results show mean ± SD from 3 wells in a single experiment.
(D,E) Summary results show $\Delta[Ca^{2+}]_c$ after restoration of extracellular Ca^{2+} to cells treated with siRNA or CcA, as paired comparisons (each with 3 replicates) and the mean value (n = 7 (D) or 3 (E), line). P = 0.18 (D) and 0.42 (E), paired Student's t-test.
(F) HEK cells pre-incubated with Baf A_1 (1 μM, 1 hr) were treated with thapsigargin (1 μM, 15 min) in Ca^{2+}-free HBS before restoration of the indicated concentrations of extracellular Ca^{2+}. Summary results show mean ± SEM, n = 3, each with 3 determinations. For clarity, only a single error bar is shown for each mean.
(G) Fluo 8-loaded HeLa cells were treated with Baf A_1 (1 μM, 1 hr) in HBS before addition of BAPTA (2.5 mM) and then histamine (100 μM) to stimulate IP_3 formation. Typical results show mean ± SD from 3 wells in a single experiment.
(H) Summary results show $\Delta[Ca^{2+}]_c$ as paired comparisons (each with 3 replicates) and the mean value (n = 5, line). $^*P < 0.05$, paired Student's t-test.
(I) HeLa cells pre-incubated with Baf A_1 (1 μM, 1 hr) were treated with thapsigargin (1 μM, 15 min) in Ca^{2+}-free HBS before restoration of extracellular Ca^{2+} (10 mM). Paired comparisons (n = 4, with 3 determinations in each) are presented in the same format as panel H (P = 0.81, paired Student's t-test).
(J) Effects of the indicated concentrations of U73122 or U73343 (20 min) on the peak increase in $[Ca^{2+}]_c$ evoked by histamine (100 μM). Results are mean ± SEM from 3 experiments, each with 3 determinations.
(K,L) Typical traces from HeLa cells expressing Ly-GG show the responses to histamine (100 μM) (K) or photolysis of ci-IP_3 (L) in Ca^{2+}-free HBS with or without U73122 (10 μM, 20 min), and then ionomycin (10 μM) with 2 mM $CaCl_2$. Results show responses of a single tracked lysosome. Summary results in **Figure 1L**.
(M) Effects of CCh on Ca^{2+} release in wild-type (WT) HEK cells and HEK cells lacking IP_3Rs (KO). Mean ± SEM, n = 3.

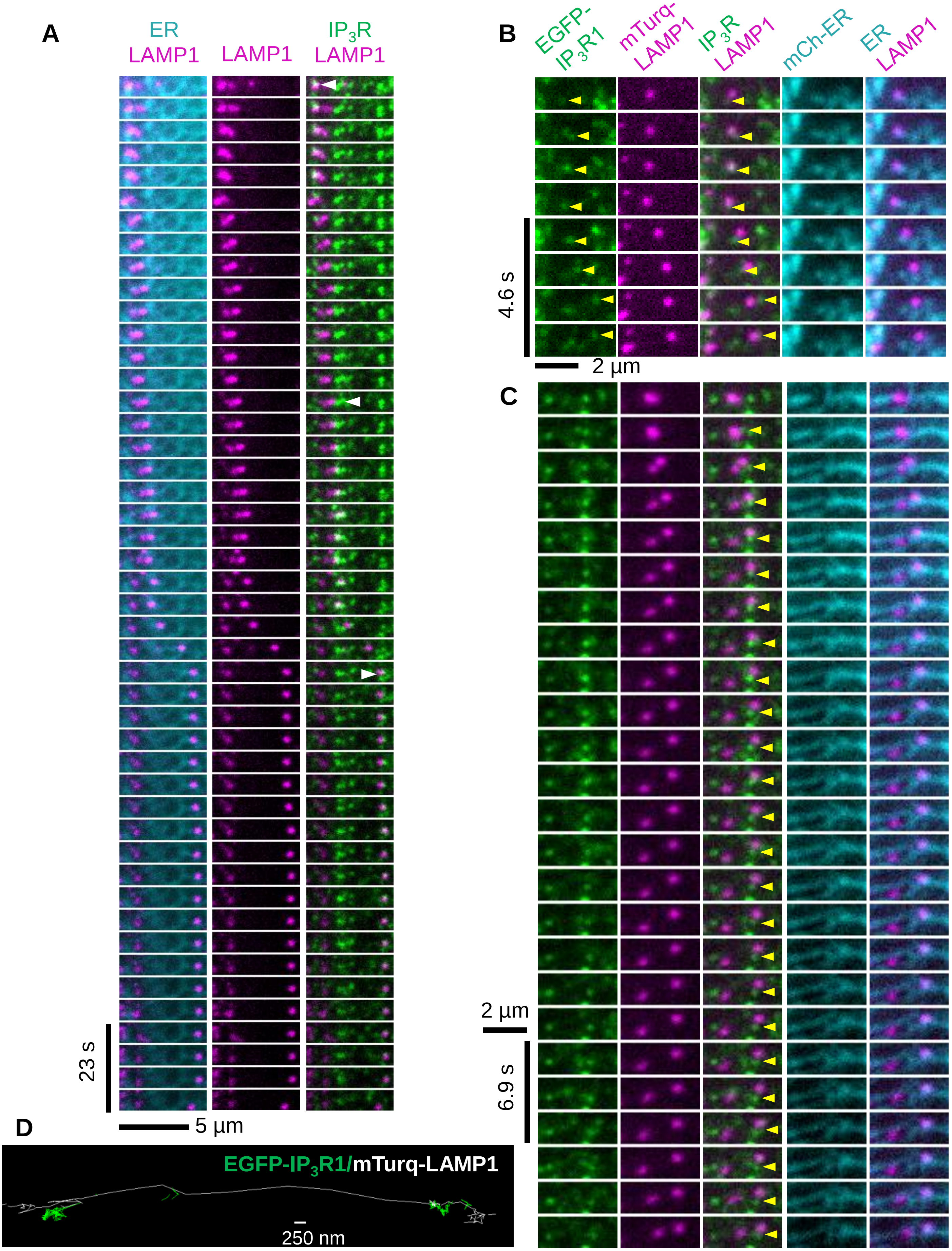

Figure S2. Lysosomes Preferentially Linger at IP$_3$R Puncta: Example 1. Related to Figures 4 and 5, Movies 5 and 6
Legend on next page

Figure S2. Lysosomes Preferentially Linger at IP_3R Puncta: Example 1

Figure on preceding page

TIRFM images of EGFP-IP_3R1-HeLa cells expressing markers of lysosomes (mTurquoise-LAMP1, pseudo-colored in magenta) and the ER lumen (mCherry-ER, pseudo-colored in cyan) were used to construct the kymograms.
(A) A lysosome moves between three different immobile IP_3R puncta (arrows), parking at each for tens of seconds, but moving rapidly along the ER between puncta (see **Movie 5**). White regions indicate colocalization of LAMP1 (magenta) and EGFP-IP_3R1 (green).
(B) A lysosome collides with an IP_3R punctum, and the two then move together. Arrows show positions of the moving IP_3R punctum.
(C) Two lysosomes separate, and one then associates with a slowly moving IP_3R punctum (arrow) with which it then moves for at least 50 s (from **Movie 6**).
(D) Single-particle trajectories (190 s) of two immobile EGFP-IP_3R1 puncta (green) and a lysosome (white) show that the lysosome pauses near the IP_3R puncta, but moves rapidly between them.

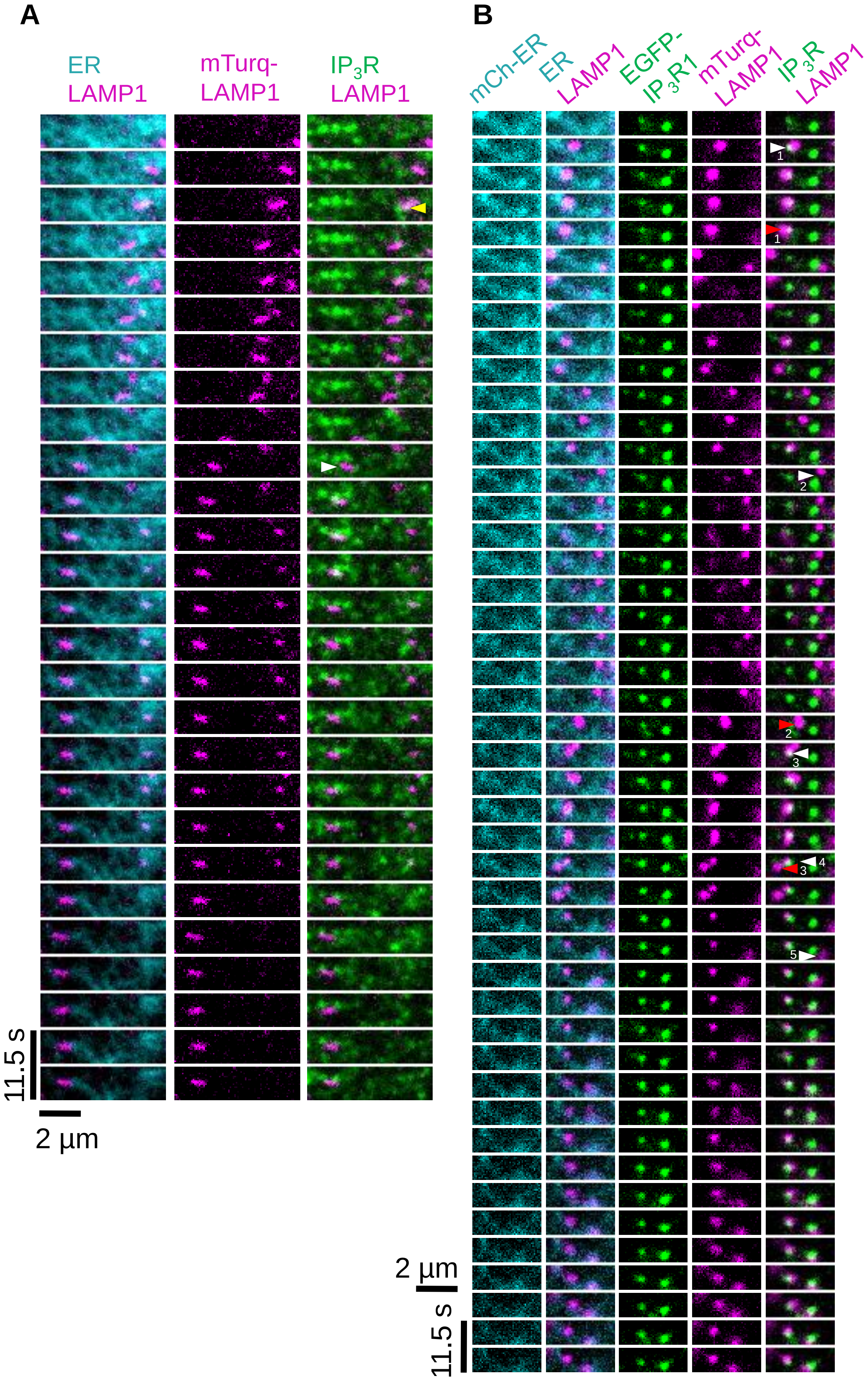

Figure S3. Lysosomes Preferentially Linger at IP_3R Puncta: Example 2. Related to Figures 4 and 5, Movies 5 and 6
Legend on next page

Figure S3. Lysosomes Preferentially Linger at IP_3R Puncta: Example 2

Figure on preceding page

TIRFM images of EGFP-IP_3R1-HeLa cells expressing markers of lysosomes (mTurquoise-LAMP1, pseudo-colored in magenta) and the ER lumen (mCherry-ER, pseudo-colored in cyan) were used to construct kymograms.
(A) Two lysosomes associated with an IP_3R punctum (yellow arrow) separate, with one leaving to join another IP_3R punctum (white arrow).
(B) Each of the two immobile IP_3R puncta shown receives several long-lasting visits by lysosomes (1-5, from **Movie 7**). Each arrival (white arrow) and departure (red arrow) is shown; encounters 4 and 5 persist beyond the recording.

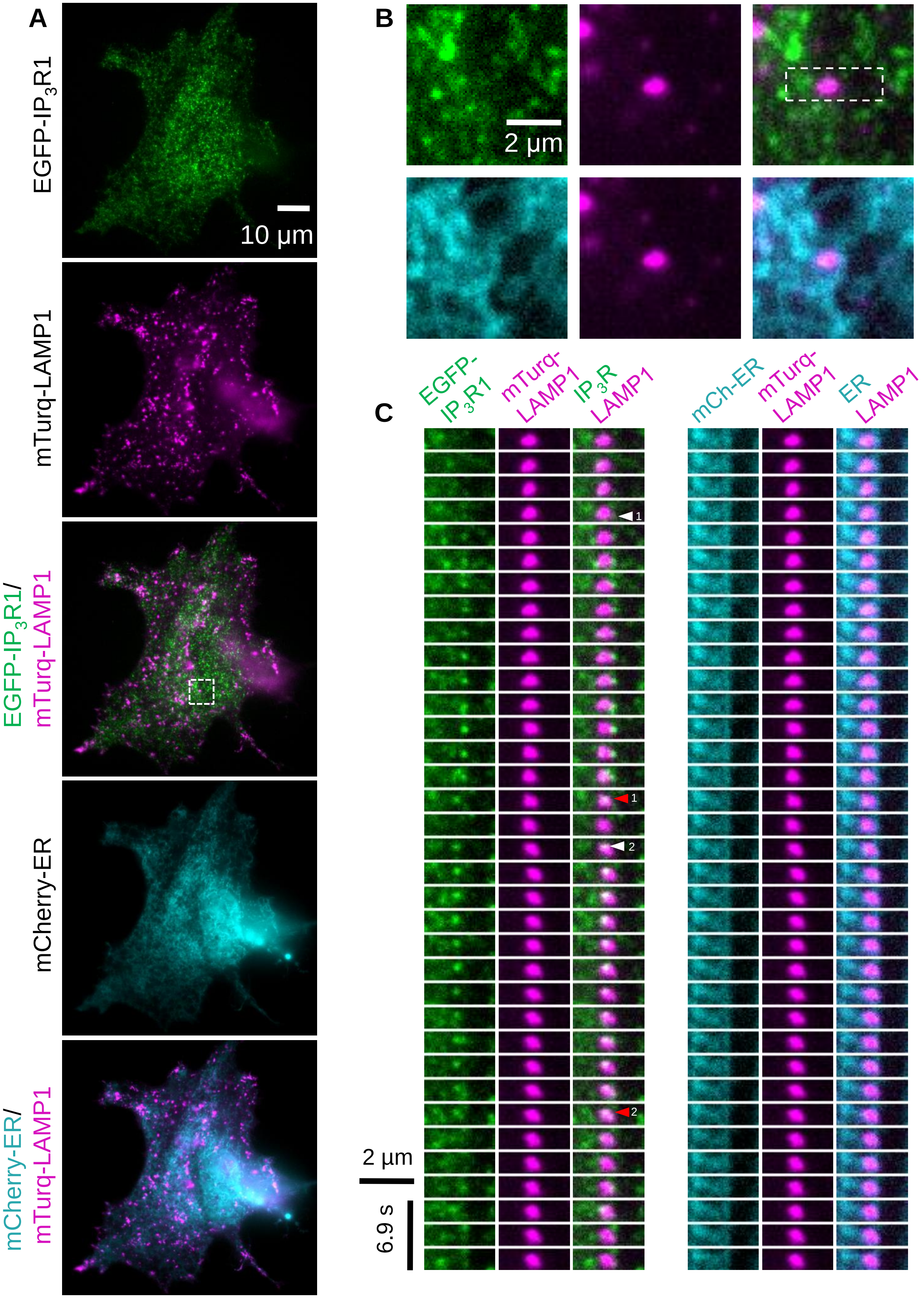

Figure S4. Several Mobile IP$_3$R Puncta Sequentially Associate With a Single Lysosome. Related to Figure 4, Movie 8
Legend on next page

Figure S4. Several Mobile IP_3R Puncta Sequentially Associate With a Single Lysosome

Figure on preceding page

(A) TIRFM images of an EGFP-IP_3R1-HeLa cell expressing markers of lysosomes (mTurquoise-LAMP1, pseudo-colored in magenta) and the ER lumen (mCherry-ER, pseudo-colored in cyan) (from **Movie 8**).
(B) Enlargements of the boxed region in panel A.
(C) Kymograms (2.3-s intervals) from boxed area in panel B show several mobile IP_3R puncta sequentially parking at a single lysosome. Arrows indicate the arrival (white) and departure (red) of two mobile IP_3R puncta.

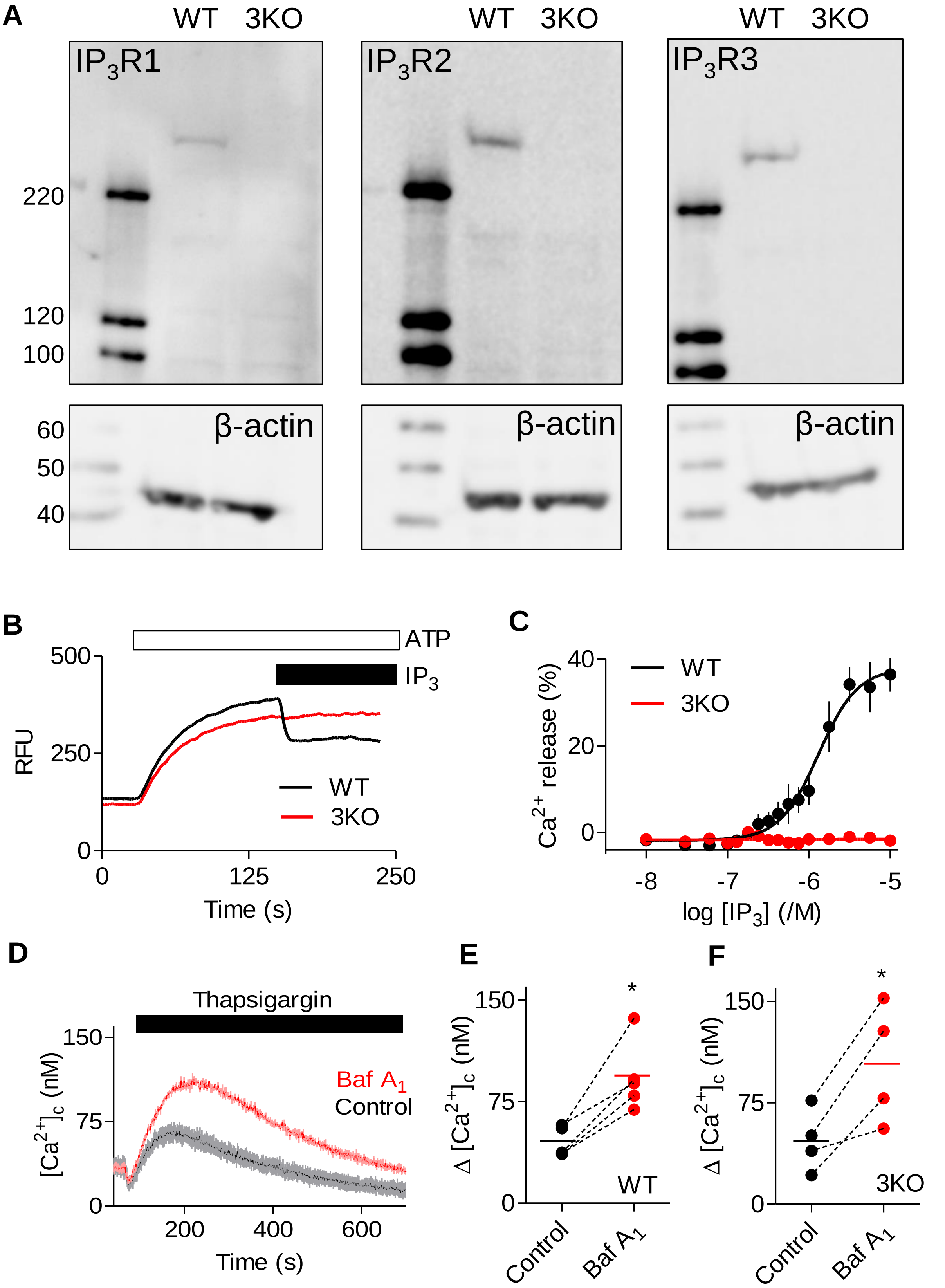

Figure S5. Bafilomycin A_1 Exaggerates Thapsigargin-Evoked Increases in $[Ca^{2+}]_c$ in HAP1 Cells Lacking IP_3Rs

Related to Figure 6, Movies 9 and 10

Legend on next page

Figure S5. Bafilomycin A_1 Exaggerates Thapsigargin-Evoked Increases in $[Ca^{2+}]_c$ in HAP1 Cells Lacking IP_3Rs

Figure on preceding page

CRISPR/Cas9 was used to disrupt the endogenous genes encoding all three IP_3R subtypes in HAP1 cells, which are a human, near-haploid, chronic myeloid leukemia cell line (Horizon Discovery, Cambridge, UK).
(A) Typical Western blots from HAP1 cells lacking all IP_3Rs (3KO) or wild-type (WT) cells, using antisera selective for each IP_3R subtype or for β-actin. M_r markers (kDa) are shown. Similar results were obtained in 3 independent analyses.
(B) Ca^{2+} uptake into the ER of saponin-permeabilized cells was recorded in cytosol-like medium, after addition of ATP, using a low-affinity Ca^{2+} indicator (Mag-fluo-4) trapped within the ER lumen (Tovey et al., 2006). The effect of IP_3 (10 μM) on Ca^{2+} release is shown for WT and 3KO cells. Typical results from a single experiment. RFU, relative fluorescence units.
(C) Summary results (mean ± SEM, n = 4) show concentration-dependent effects of IP_3 on Ca^{2+} release.
(D) HAP1 cells were treated with bafilomycin A_1 (Baf A_1, 1 μM, 1 hr) in HBS before addition of BAPTA (2.5 mM) to chelate extracellular Ca^{2+} and then thapsigargin (1 μM) to inhibit SERCA. Typical results show mean ± SD from 3 wells in a single experiment.
(E,F) Summary results show peak thapsigargin-evoked increase in $[Ca^{2+}]_c$ in WT (E) and 3KO cells (F) as paired observations (each with 3 determinations) and the mean value (n = 5 (E), 4 (F), line). $^*P < 0.05$, paired Student's t-test.

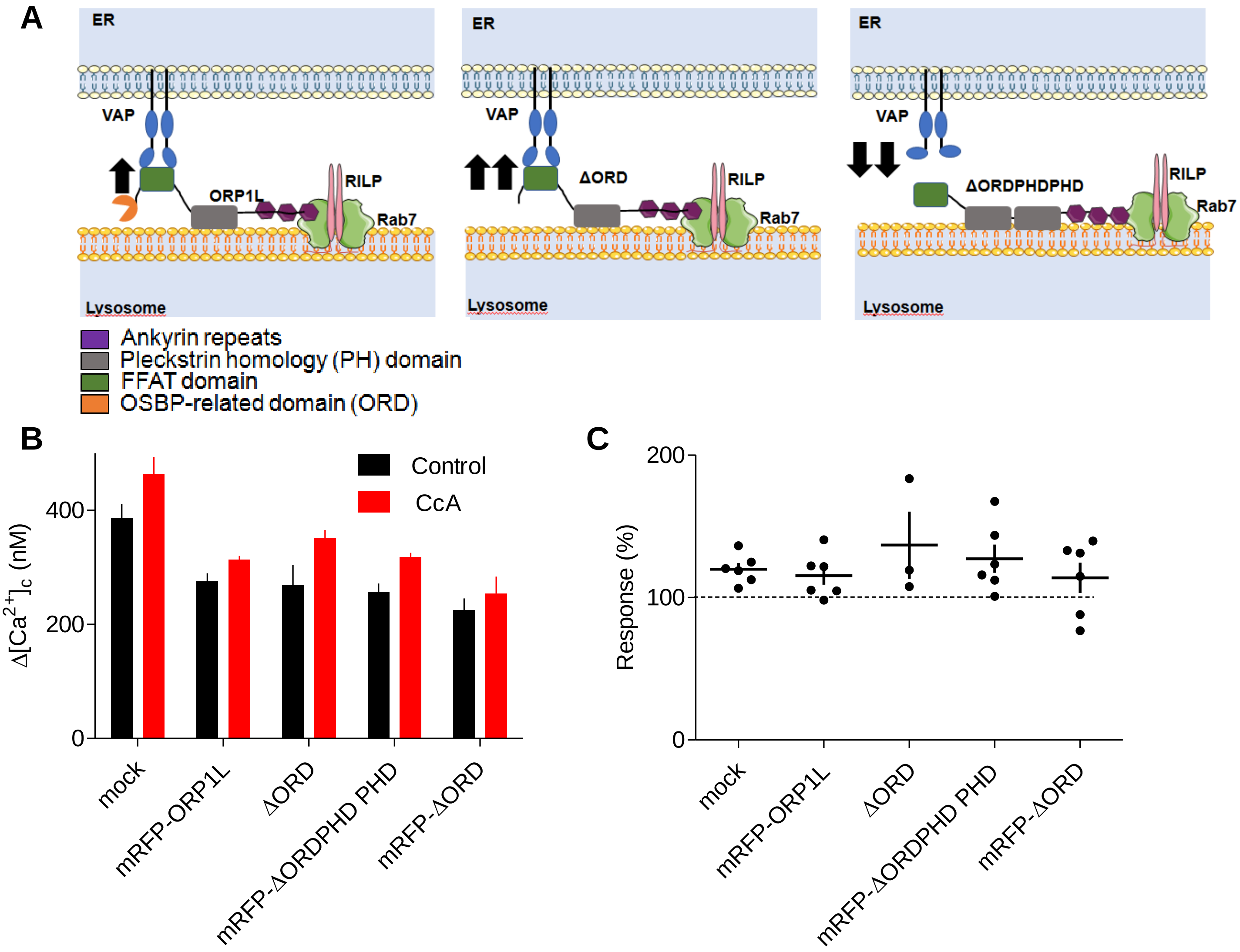

Figure S6. ORP1L is Unlikely to Contribute to ER-Lysosome MCS at which Ca^{2+} Exchange Occurs Related to Figures 4-6

(A) ORP1L associated with rab7 in the lysosome membrane stabilizes ER-lysosome MCS by interacting, through its FFAT domain, with VAP in the ER (Rocha et al., 2009). Cholesterol, by binding to the ORD domain, disrupts the interaction. Hence ORP1L without the ORD domain (ΔORD) forms more stable junctions. The PH domain of ORPL1 binds to the lysosome membrane. Hence, duplicating the domain (ΔORDPHDPHD) destabilizes the MCS.
(B) HEK cells transiently transfected to express ORP1L proteins were stimulated in Ca^{2+}-free HBS with CCh (1 mM) alone or after treatment with CcA (1 µM, 1 hr). Results (mean ± SEM, n = 6 (except for ΔORD, n = 3), each with 3 replicates) show peak increase in $[Ca^{2+}]_c$ evoked by CCh.
(C) For each paired analysis, $\Delta[Ca^{2+}]_c$ evoked by CCh in the presence of CcA was expressed as a percentage of the response to CCh alone (100%).

Our attempts to use siRNA to assess the contributions of ORP1L to ER-lysosome Ca^{2+} exchange were frustrated by the ineffectiveness of two different siRNAs and by the existence of two ORP1 variants. The siRNAs reduced expression of the 55-kDa band by ~17%, but had no significant effect on expression of the 120-kDa band (Johansson et al., 2003).

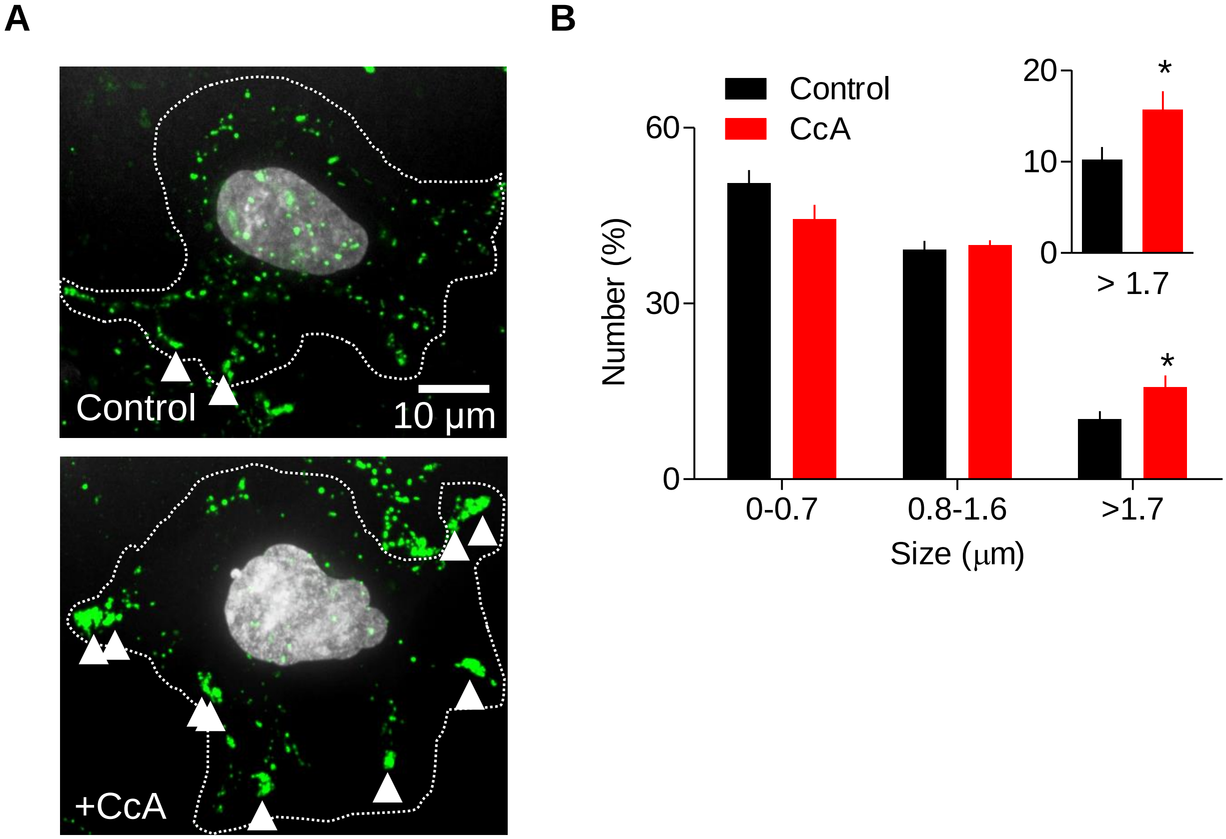

Figure S7. Concanamycin A Causes Redistribution and Enlargement of Lysosomes
Related to Figure 7

(A) Confocal section (close to the coverslip) of HEK cell loaded with Alexa Fluor 488-dextran (10,000 MW) (green) with and without treatment with concanamycin A (CcA, 1 μM, 1 hr). Nuclei, stained with NucBlue are shown in grey. The outline of a single cell is shown. Arrows highlight examples of enlarged lysosomes. Scale bar applies to both images.
(B) Summary results show lysosomes (%) categorized by their Feret diameter (see STAR METHODS). Results are from 653 (control) and 893 (CcA-treated) lysosomes from 7 cells in 3 independent experiments. Inset shows enlargement of the largest size category. $^{*}P < 0.05$, Student's *t*-test, relative to control.
