# Supplementary material for: IP3 Receptors Preferentially Associate with ER-Lysosome Contact Sites and Selectively Deliver Ca2+ to Lysosomes
Source: Cell Rep. 2018 Dec 11;25(11):3180–3193.e7. doi: 10.1016/j.celrep.2018.11.064 (PMC6302550; doi:10.1016/j.celrep.2018.11.064)
Supplement: Document S2. Article plus Supplemental Information [file mmc12.pdf]

Cell Reports

Article

IP_3 Receptors Preferentially Associate with ER-Lysosome Contact Sites and Selectively Deliver Ca^{2+} to Lysosomes

Graphical Abstract

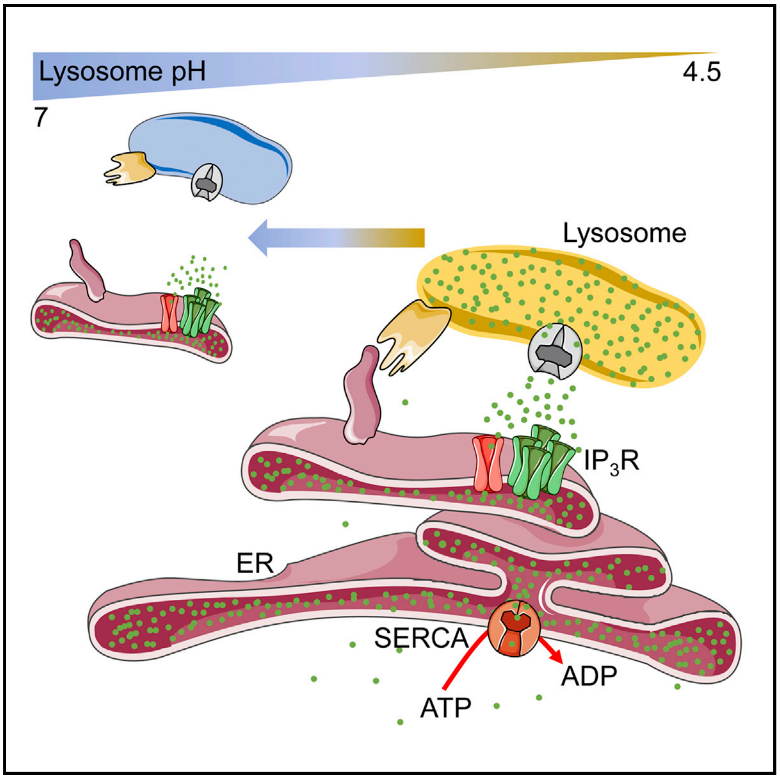

Authors

Peace Atakpa,
Nagendra Babu Thillaiappan,
Stefania Mataragka, David L. Prole,
Colin W. Taylor

Correspondence

cwt1000@cam.ac.uk

In Brief

Ca^{2+} exchanges between ER and lysosomes regulate cytosolic Ca^{2+} signals and lysosome behavior. Atakpa et al. show that clusters of IP_3 receptors populate ER-lysosome contact sites and facilitate local delivery of Ca^{2+} from the ER to lysosomes.

Highlights

- IP_3 receptors (IP_3Rs) selectively deliver Ca^{2+} to lysosomes
- Lysosomes associate preferentially with clusters of IP_3Rs in ER membranes
- Low lysosomal pH maintains the IP_3R-lysosome contacts required for Ca^{2+} uptake
- ER and its Ca^{2+} channels deliver Ca^{2+} to low-affinity lysosomal transporters

Atakpa et al., 2018, Cell Reports *25*, 3180–3193
December 11, 2018 © 2018 The Author(s).
https://doi.org/10.1016/j.celrep.2018.11.064

CellPress

IP_3 Receptors Preferentially Associate with ER-Lysosome Contact Sites and Selectively Deliver Ca^{2+} to Lysosomes

Peace Atakpa,[1] Nagendra Babu Thillaiappan,[1] Stefania Mataragka,[1] David L. Prole,[1] and Colin W. Taylor[1,2,*]
[1]Department of Pharmacology, University of Cambridge, Tennis Court Road, Cambridge CB2 1PD, UK
[2]Lead Contact
*Correspondence: cwt1000@cam.ac.uk
https://doi.org/10.1016/j.celrep.2018.11.064

SUMMARY

Inositol 1,4,5-trisphosphate (IP_3) receptors (IP_3Rs) allow extracellular stimuli to redistribute Ca^{2+} from the ER to cytosol or other organelles. We show, using small interfering RNA (siRNA) and vacuolar H^+-ATPase (V-ATPase) inhibitors, that lysosomes sequester Ca^{2+} released by all IP_3R subtypes, but not Ca^{2+} entering cells through store-operated Ca^{2+} entry (SOCE). A low-affinity Ca^{2+} sensor targeted to lysosomal membranes reports large, local increases in cytosolic $[Ca^{2+}]$ during IP_3-evoked Ca^{2+} release, but not during SOCE. Most lysosomes associate with endoplasmic reticulum (ER) and dwell at regions populated by IP_3R clusters, but IP_3Rs do not assemble ER-lysosome contacts. Increasing lysosomal pH does not immediately prevent Ca^{2+} uptake, but it causes lysosomes to slowly redistribute and enlarge, reduces their association with IP_3Rs, and disrupts Ca^{2+} exchange with ER. In a "piston-like" fashion, ER concentrates cytosolic Ca^{2+} and delivers it, through large-conductance IP_3Rs, to a low-affinity lysosomal uptake system. The involvement of IP_3Rs allows extracellular stimuli to regulate Ca^{2+} exchange between the ER and lysosomes.

INTRODUCTION

Increases in cytosolic free Ca^{2+} concentration ($[Ca^{2+}]_c$) regulate the activities of all cells, allowing them to respond to internal and extracellular signals. Most Ca^{2+} signals are evoked by opening of Ca^{2+}-permeable channels within the plasma membrane (PM) or the membranes of intracellular organelles, usually the endoplasmic reticulum (ER). In non-excitable cells, extracellular stimuli typically evoke Ca^{2+} signals by stimulating phospholipase C (PLC), which catalyzes formation of inositol 1,4,5-trisphosphate (IP_3). Binding of both IP_3 and Ca^{2+} to IP_3 receptors (IP_3Rs) causes them to open and release Ca^{2+} from the ER (Foskett et al., 2007; Taylor and Tovey, 2010).

The spatial organization of Ca^{2+} signals allows Ca^{2+} entering the cytosol through different channels to evoke different responses (Giorgi et al., 2018). Mitochondria, for example, when juxtaposed to ER, selectively sequester Ca^{2+} released by IP_3Rs, and this then regulates mitochondrial behavior (Rizzuto et al., 2012). Ca^{2+} released through IP_3Rs or ryanodine receptors (RyR) adjacent to the PM regulates membrane potential by selectively activating Ca^{2+}-sensitive Cl^- or K^+ channels (Courjaret et al., 2017; Nelson et al., 1995). Depolarization of cardiac muscle opens voltage-gated Ca^{2+} channels, and the resulting local Ca^{2+} signals are amplified by Ca^{2+}-induced Ca^{2+} release (CICR) from RyRs (Ríos, 2018). Loss of Ca^{2+} from the ER stimulates Ca^{2+} channels in the PM, and the resulting store-operated Ca^{2+} entry (SOCE) selectively regulates adenylyl cyclases, nitric oxide synthase, and nuclear factor of activated T cells (Prakriya and Lewis, 2015). For each of these examples, and many others, the specificity of the Ca^{2+} signal is conferred by having a channel deliver Ca^{2+} at a high local concentration to closely apposed target proteins.

Lysosomes can also sequester Ca^{2+} and they express channels, including TRPML (transient receptor potential mucolipin), TPC2 (two-pore channel 2), and ATP-regulated $P2X_4$ receptors, that allow Ca^{2+} release (Morgan et al., 2011). Here, too, cross-talk with the ER is important, and it is facilitated by membrane contact sites (MCSs) between lysosomes and ER, stabilized by scaffold proteins (Alpy et al., 2013; Eden, 2016; Friedman et al., 2013; Kilpatrick et al., 2017). The cytosolic Ca^{2+} signals evoked by TRPML or TPC2 can be amplified by CICR through IP_3Rs or RyRs in closely apposed ER (Galione, 2015; Morgan et al., 2011; Patel et al., 2010). Conversely, Ca^{2+} released by ER channels can be rapidly sequestered by lysosomes. This sequestration attenuates cytosolic Ca^{2+} signals evoked by IP_3Rs (López Sanjurjo et al., 2013) and, by loading lysosomes with Ca^{2+}, primes TPC2 to respond (Morgan et al., 2013), controls fusion and fission within endolysosomal pathways (Ruas et al., 2010), and regulates autophagy and lysosomal biogenesis through calcineurin activated by TRPML-mediated Ca^{2+} release (Medina et al., 2015). Using pharmacological inhibitors that disrupt lysosomes, perturb their morphology, or block their ability to sequester H^+, we showed previously that the increase in $[Ca^{2+}]_c$ evoked by IP_3Rs was exaggerated when lysosomes were disrupted, but SOCE-evoked Ca^{2+} signals were unaffected (López Sanjurjo et al., 2013, 2014). We suggested that lysosomes selectively sequester Ca^{2+} released by IP_3Rs, while ignoring Ca^{2+} entering cells through SOCE.

Using targeted low-affinity Ca^{2+} sensors, we now show that IP_3Rs selectively deliver Ca^{2+} to lysosomes. Many long-lived contacts between ER and lysosomes are populated by small

 © 2018 The Author(s).
This is an open access article under the CC BY license (http://creativecommons.org/licenses/by/4.0/).

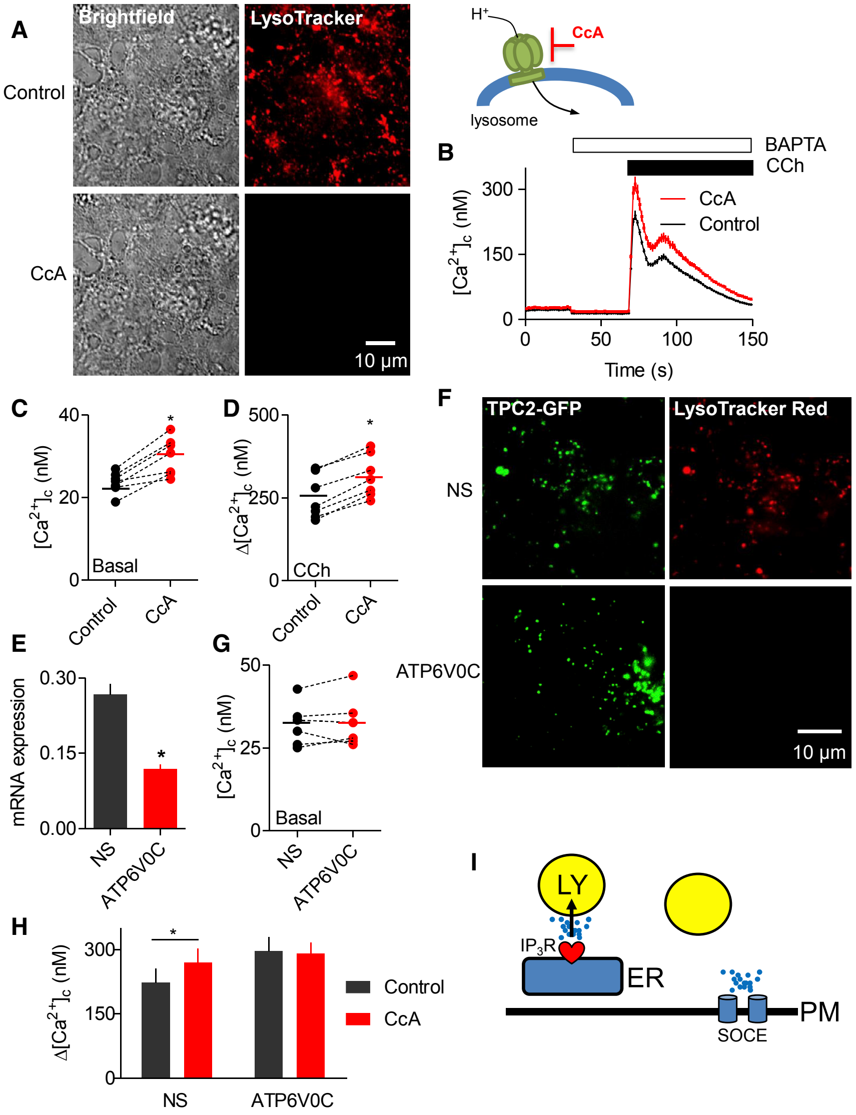

Figure 1. Inhibition of Lysosomal V-ATPase Potentiates Cytosolic Ca^{2+} Signals Evoked by IP_3Rs

(A) Bright-field and wide-field fluorescence images of HEK cells loaded with LysoTracker Red (100 nM, 10 min) with or without CcA (1 μM, 1 hr). Images are typical of three experiments.

(B) Fluo 8-loaded HEK cells were treated with CcA (1 μM, 1 hr) in HBS before addition of 1,2-bis (o-aminophenoxy)ethane-*N,N,N′,N′*-tetraacetic acid (BAPTA) (2.5 mM) to chelate extracellular Ca^{2+} and then CCh (1 mM) to stimulate IP_3 formation. Typical traces show mean ± SD from three wells in one experiment.

(C and D) Summary results show effects of CcA on basal $[Ca^{2+}]_c$ (C) and peak increase in $[Ca^{2+}]_c$ ($\Delta[Ca^{2+}]_c$) evoked by CCh (D). Results show paired individual values (each from three determinations) and the mean (n = 7, line). $^*p < 0.05$, paired Student's t test.

(E) Expression of mRNA for ATP6V0C relative to GAPDH in cells treated with non-silencing siRNA (NS) or siRNA for ATP6V0C. Mean ± SEM, n = 6. $^*p < 0.05$, paired Student's t test.

(F) TIRFM images show effects of siRNAs in HEK cells expressing TPC2-GFP or stained with LysoTracker Red (100 nM, 10 min). Images are typical of three experiments.

(G) Effects of siRNA on basal $[Ca^{2+}]_c$ (n = 6, each with three determinations).

(H) Effects of siRNA on $\Delta[Ca^{2+}]_c$ evoked by CCh alone or after CcA (1 μM, 1 hr) (mean ± SEM, n = 5, each with three determinations). $^*p < 0.05$, two-way ANOVA with Bonferroni test.

(I) Results and Figure S1 demonstrate that lysosomes (LY) selectively sequester Ca^{2+} released from ER through IP_3Rs, but not Ca^{2+} entering the cell through SOCE.

See also Figure S1.

clusters of IP_3Rs. Increasing lysosomal pH does not immediately prevent Ca^{2+} uptake, but it slowly causes lysosomes to enlarge, redistribute, reduce their affiliation with IP_3Rs, and lose their ability to selectively sequester Ca^{2+} released by IP_3Rs. We conclude that the ER, with its IP_3Rs and high-affinity Ca^{2+} pump (SERCA, sarcoplasmic/endoplasmic reticulum Ca^{2+}-ATPase), can, in "piston-like" fashion, deliver Ca^{2+} from the cytosol with its low $[Ca^{2+}]_c$ to the low-affinity uptake system of lysosomes. The involvement of IP_3Rs allows cell-surface receptors, through PLC and IP_3, to regulate this Ca^{2+} transfer and so the behavior of lysosomes.

RESULTS

Lysosomes Selectively Sequester Ca^{2+} Released by IP_3Rs

The vacuolar H^+-ATPase (V-ATPase) maintains the luminal pH of lysosomes at about 4.5. Treatment of HEK cells with concanamycin A (CcA), a more selective inhibitor of the V-ATPase than bafilomycin A_1 (Dröse et al., 1993), dissipated the lysosomal pH gradient (Figure 1A) and modestly increased the basal $[Ca^{2+}]_c$ (Figures 1B and 1C). The peak increase in $[Ca^{2+}]_c$ evoked by carbachol (CCh), which stimulates IP_3 formation by activating M_3 muscarinic receptors in HEK cells, was increased by CcA (Figures 1B and 1D). Similar results were obtained with bafilomycin A_1 (Figures S1A and S1B) (López Sanjurjo et al., 2013).

A small interfering RNA (siRNA) to an essential pore-forming subunit of the V-ATPase (ATP6V0C) (Forgac, 2007) reduced expression of its mRNA (Figure 1E). There are no reliable antibodies to determine expression of ATP6V0C protein (Mangieri et al., 2014). Reduced expression of ATP6V0C caused lysosomes to enlarge, and it increased the pH within them, determined using LysoTracker Red (Figure 1F), without significantly affecting the basal $[Ca^{2+}]_c$ (Figure 1G). However, siRNA to ATP6V0C increased the amplitude of the CCh-evoked Ca^{2+} signals to the same extent as CcA, and there was no further effect of CcA on the CCh-evoked increase in $[Ca^{2+}]_c$ after knockdown of the V-ATPase (Figure 1H). These results confirm that the

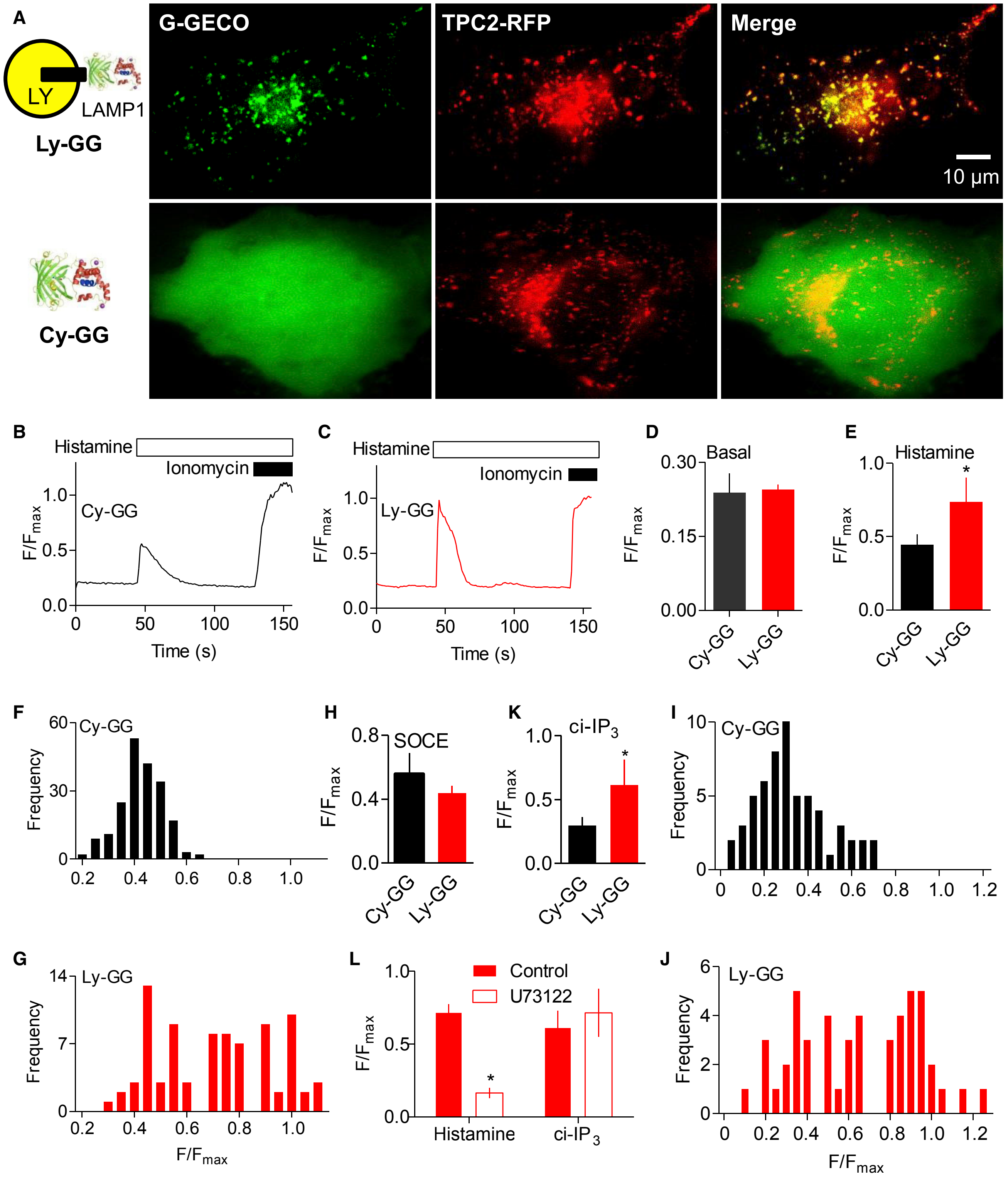

Figure 2. IP_3Rs Selectively Deliver Ca^{2+} to Some Lysosomes

(A) Wide-field fluorescence images of HeLa cells expressing TPC2-RFP with Ly-GG or Cy-GG.

(B and C) Recordings from HeLa cells expressing Cy-GG (B) or Ly-GG (C) showing responses to histamine (100 μM) in Ca^{2+}-free HBS and then ionomycin (10 μM) with 2 mM $CaCl_2$ (to saturate the sensor). Results (F/F_{max}, where F_{max} is response after ionomycin) show responses of a single tracked lysosome (C) or a similarly sized cytosolic region of interest (ROI) (B) (see Video S1).

(legend continued on next page)

effects of CcA on CCh-evoked Ca^{2+} signals are mediated by inhibition of the V-ATPase. The results are important because bafilomycin A_1 and CcA have additional effects, which at higher concentrations include inhibition of the P-type ATPases that transport Ca^{2+} across the PM and ER membranes (Dröse et al., 1993). We conclude that inhibition of the V-ATPase potentiates the increase in $[Ca^{2+}]_c$ evoked by Ca^{2+} release through IP_3Rs.

The sustained response to CCh, and to most stimuli that activate PLC, requires Ca^{2+} entry across the PM through SOCE, which is stimulated when IP_3 causes loss of Ca^{2+} from the ER (López Sanjurjo et al., 2014; Prakriya and Lewis, 2015). In cells pretreated with thapsigargin in Ca^{2+}-free HEPES-buffered saline (HBS) to inhibit SERCA and deplete the ER of Ca^{2+}, restoration of extracellular Ca^{2+} caused a sustained increase in $[Ca^{2+}]_c$ reflecting the activity of SOCE (Figure S1C). Although the global increase in $[Ca^{2+}]_c$ resulting from thapsigargin-evoked SOCE was comparable with the increase after CCh-evoked Ca^{2+} release (Figures 1B and S1C), SOCE signals were unaffected by CcA, bafilomycin A_1, or knockdown of the V-ATPase (Figures S1D–S1F).

These results extend previous observations (López Sanjurjo et al., 2013) by demonstrating that dissipating the lysosomal pH gradient, using siRNA to the V-ATPase or pharmacological inhibitors, exaggerates cytosolic Ca^{2+} signals evoked by IP_3Rs, but not those evoked by SOCE (Figure 1I).

IP_3Rs Selectively Deliver Ca^{2+} to Lysosomes

The conclusion that Ca^{2+} released from the ER is selectively accumulated by lysosomes has so far been inferred from exaggerated increases in $[Ca^{2+}]_c$ after perturbing lysosomes (Figures 1 and S1A–S1I). We attempted to provide direct evidence, free of these perturbations, using a low-affinity Ca^{2+} sensor (G-GECO1.2, K_D^{Ca} = 1.2 μM) (Zhao et al., 2011) targeted to the cytosolic surface of lysosomes by attaching it to LAMP1 (Ly-GG). For comparison with these measurements of $[Ca^{2+}]$ near lysosomal membranes, we used the same sensor expressed in the cytosol (Cy-GG) to record global increases in $[Ca^{2+}]_c$ (Video S1). HeLa cells, in which histamine stimulates IP_3 formation and Ca^{2+} release from the ER (Thillaiappan et al., 2017), were used for these experiments because they are better suited for imaging organelles. In HeLa cells, just as in HEK cells, IP_3-evoked increases in $[Ca^{2+}]_c$ were potentiated by bafilomycin A_1, whereas SOCE-evoked Ca^{2+} signals were not (Figures S1G–S1I). Because the peak $[Ca^{2+}]_c$ after histamine stimulation does not exceed ~360 nM (Figure S1G), Ly-GG and Cy-GG selectively report local increases in $[Ca^{2+}]_c$ in HeLa cells.

Ly-GG co-localized with LAMP1-mCh (R_{coloc} = 0.93 ± 0.02, n = 3; R_{coloc} is Pearson's correlation coefficient) and with the lysosomal channel, TPC2-RFP (R_{coloc} = 0.86 ± 0.09, n = 3) (Figure 2A). In unstimulated cells, $[Ca^{2+}]_c$ (reported as F/F_{max}) was similar when detected with Ly-GG or with Cy-GG (Figures 2B–2D). This confirms that the Ca^{2+}-affinity of the sensor was unaffected by differential targeting. Cells expressing the Ca^{2+} sensor and incubated in Ca^{2+}-free HBS were stimulated with histamine to evoke IP_3 formation. Fluorescence was recorded from either single-tracked lysosomes (Ly-GG) or from comparable areas of cytosol (Cy-GG). Histamine caused a transient increase in Cy-GG fluorescence and a larger transient increase in Ly-GG fluorescence; the latter often came close to saturating the sensor (Figures 2C and 2E). From the distribution of fluorescence intensity changes of Ly-GG from 83 tracked lysosomes, 41% of responses overlapped those recorded from Cy-GG, but the remaining 59% of lysosomes responded with much larger fluorescence changes (Figures 2F and 2G). There was no difference in the average speed of lysosomes responding to histamine with large (F/F_{max} > 0.6; speed = 0.45 ± 0.17 μm/s, n = 51) or cytosol-like responses (F/F_{max} < 0.6; 0.34 ± 0.22 μm/s, n = 32; p = 0.60). Similar analyses of SOCE revealed no disparity in the responses of Cy-GG and Ly-GG: both sensors reported similar increases in $[Ca^{2+}]_c$ (Figure 2H).

We considered whether near-lysosome Ca^{2+} signals might be due to lysosomal Ca^{2+} channels, rather than to juxtaposed IP_3Rs. H_1 receptors activated by histamine can, for example, stimulate accumulation of NAADP, which evokes Ca^{2+} release through TPC2 (Galione, 2015). However, two lines of evidence show that Ca^{2+} release through IP_3Rs is required for the near-lysosome Ca^{2+} signals. First, U73122, an inhibitor of PLC, abolished the histamine-evoked increases in $[Ca^{2+}]_c$, whereas the inactive analog, U73343, did not (Figure S1J). U73122 also abolished histamine-evoked Ly-GG signals (Figures 2L and S1K). Second, direct activation of IP_3Rs by photolysis of caged IP_3 (ci-IP_3) also caused increases in $[Ca^{2+}]_c$ that were larger at the lysosome surface than in bulk cytosol (Figures 2I and 2J). Furthermore, whereas U73122 abolished Ly-GG responses to histamine, it had no effect on responses to photolysis of ci-IP_3 (Figures 2L, S1K, and S1L), consistent with the effects of U73122 on histamine-evoked Ca^{2+} signals arising from inhibition of PLC.

(D and E) Summary results (mean ± SEM) show basal fluorescence (D) (3 experiments with 198 ROIs for Cy-GG and 83 tracks for Ly-GG) and peak fluorescence signals evoked by histamine (E) for Cy-GG (3 experiments with 198 ROIs) and Ly-GG (4 experiments with 83 lysosome tracks). *p < 0.05, Student's t test.

(F and G) Distribution of peak F/F_{max} values for Cy-GG (F) and Ly-GG (G) in cells stimulated with histamine. Distribution of Ly-GG fluorescence values is significantly different from a normal distribution (Kolmogorov-Smirnov normality test, p = 0.0018), whereas Cy-GG fluorescence is consistent with a normal distribution (p > 0.1).

(H) Peak responses from tracked regions for Cy-GG and Ly-GG for cells in which SOCE was evoked by restoration of extracellular Ca^{2+} (2 mM) to cells treated with thapsigargin (1 μM, 15 min) in Ca^{2+}-free HBS. Mean ± SEM from at least three experiments (30 tracks for Ly-GG and 45 ROIs for Cy-GG).

(I and J) Distribution of peak F/F_{max} values for Cy-GG (I) and Ly-GG (J) after photolysis of ci-IP_3 in Ca^{2+}-free HBS (n = 59 ROIs from 4 dishes for Cy-GG and 49 tracks from 3 dishes for Ly-GG).

(K) Summary results (mean ± SEM). *p < 0.05, Student's t test.

(L) Effects of U73122 (10 μM, 20 min) on peak Ly-GG signals evoked by histamine (100 μM) or photolysis of ci-IP_3. Mean ± SEM, n = 3–4. *p < 0.05, Student's t test, relative to control.

See also Figure S1 and Video S1.

OPEN ACCESS
CellPress

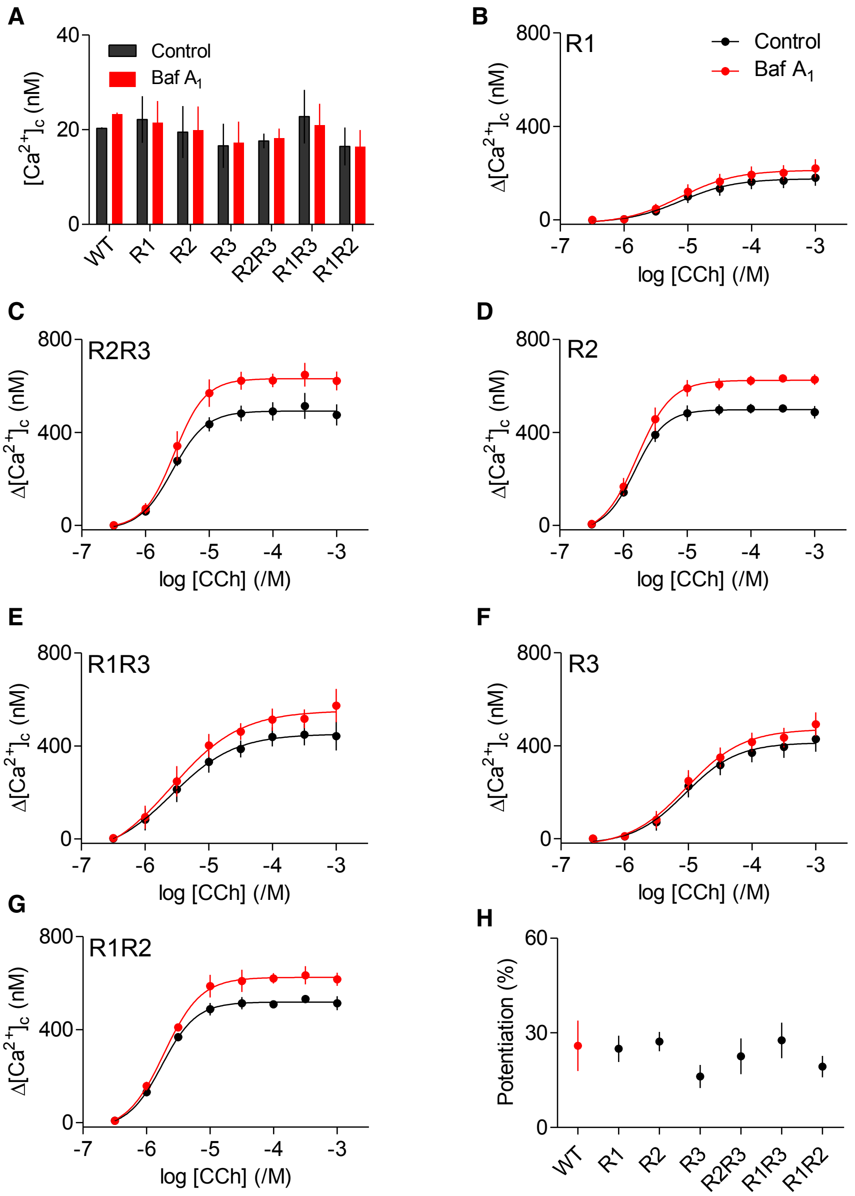

Figure 3. Lysosomes Sequester Ca^{2+} Released by All IP_3R Subtypes

(A) Basal $[Ca^{2+}]_c$ in HEK cells expressing only the indicated IP_3R subtypes and treated with bafilomycin A_1 (Baf A_1, 1 μM, 1 hr). Mean ± SEM. n = 3, each with three determinations.

(B–G) Effects of Baf A_1 (1 μM, 1 hr) on Ca^{2+} release evoked by CCh in HEK cells expressing only the indicated IP_3R subtypes. Mean ± SEM, *n* = 6. The code in (B) applies also to (C)–(G). Similar results from WT cells are shown in Figure S1B.

(H) Summary results show the potentiating effect of Baf A_1 on the peak CCh-evoked Ca^{2+} signal. Results (mean ± SD) show the increase in amplitude of Ca^{2+} signal in the presence of Baf A_1 as a percentage of the control response.

WT, wild-type. See also Figure S1.

These results show that during Ca^{2+} release from IP_3Rs, about 60% of lysosomes experience a much larger increase in $[Ca^{2+}]_c$ than the global increase. SOCE, by contrast, does not deliver Ca^{2+} to lysosomes. We conclude that Ca^{2+} is selectively delivered to lysosomes by IP_3Rs, but not SOCE (Figure 1I).

Lysosomes Sequester Ca^{2+} Released through All Three IP_3R Subtypes

The Ca^{2+} signals evoked by histamine in HeLa cells or by CCh in HEK cells are initiated by IP_3Rs, consistent with the lack of response to CCh in HEK cells without IP_3Rs (Figure S1M). The HEK cells used express all three IP_3R subtypes (IP_3R3 > IP_3R1 > IP_3R2) (Mataragka and Taylor, 2018). We also used HEK cells where genes for one or two of the three IP_3R subtypes were disrupted (Alzayady et al., 2016) to establish whether any IP_3R subtype selectively presents Ca^{2+} to lysosomes. There was no significant difference in the basal $[Ca^{2+}]_c$ between the seven cell lines examined, nor did bafilomycin A_1 affect basal $[Ca^{2+}]_c$ in any of the cells (Figure 3A). There were some unexpected differences in the amplitudes of the increase in $[Ca^{2+}]_c$ evoked by a maximal concentration of CCh (1 mM) between wild-type cells (Figure S1B) and cells lacking one or more IP_3R subtypes, with some of the latter giving larger signals than wild-type cells (Figures 3B–3G). We have not explored this further; it may arise from changes in expression of M_3 receptors or downstream signaling proteins during selection of cell lines. However, the effects of bafilomycin A_1 were similar in wild-type HEK cells and in cells lacking any one or two of the native IP_3R subtypes, irrespective of the amplitude of the CCh-evoked Ca^{2+} signals (Figures 3B–3G). In each case, bafilomycin A_1 caused the increase in $[Ca^{2+}]_c$ evoked by CCh to increase by about 20%–30% (Figure 3H). These results demonstrate that all three IP_3R subtypes can deliver Ca^{2+} to lysosomes.

IP_3Rs and Lysosomes Preferentially Associate

In COS-7 cells, most lysosomes detected near the PM by total internal reflection fluorescence microscopy (TIRFM) are associated with ER, and lysosomes maintain these associations as they move (López Sanjurjo et al., 2013). This is consistent with evidence from other cells showing that many lysosomes form dynamic MCSs with the ER (Friedman et al., 2013; Garrity et al., 2016; Kilpatrick et al., 2017). There is a similar relationship between the ER and lysosomes in HeLa cells (Figure 4A; Videos S2 and S3). We used HeLa cells in which endogenous IP_3R1 had been tagged by gene editing with EGFP (EGFP-IP_3R1-HeLa cells) (Thillaiappan et al., 2017) to explore dynamic relationships

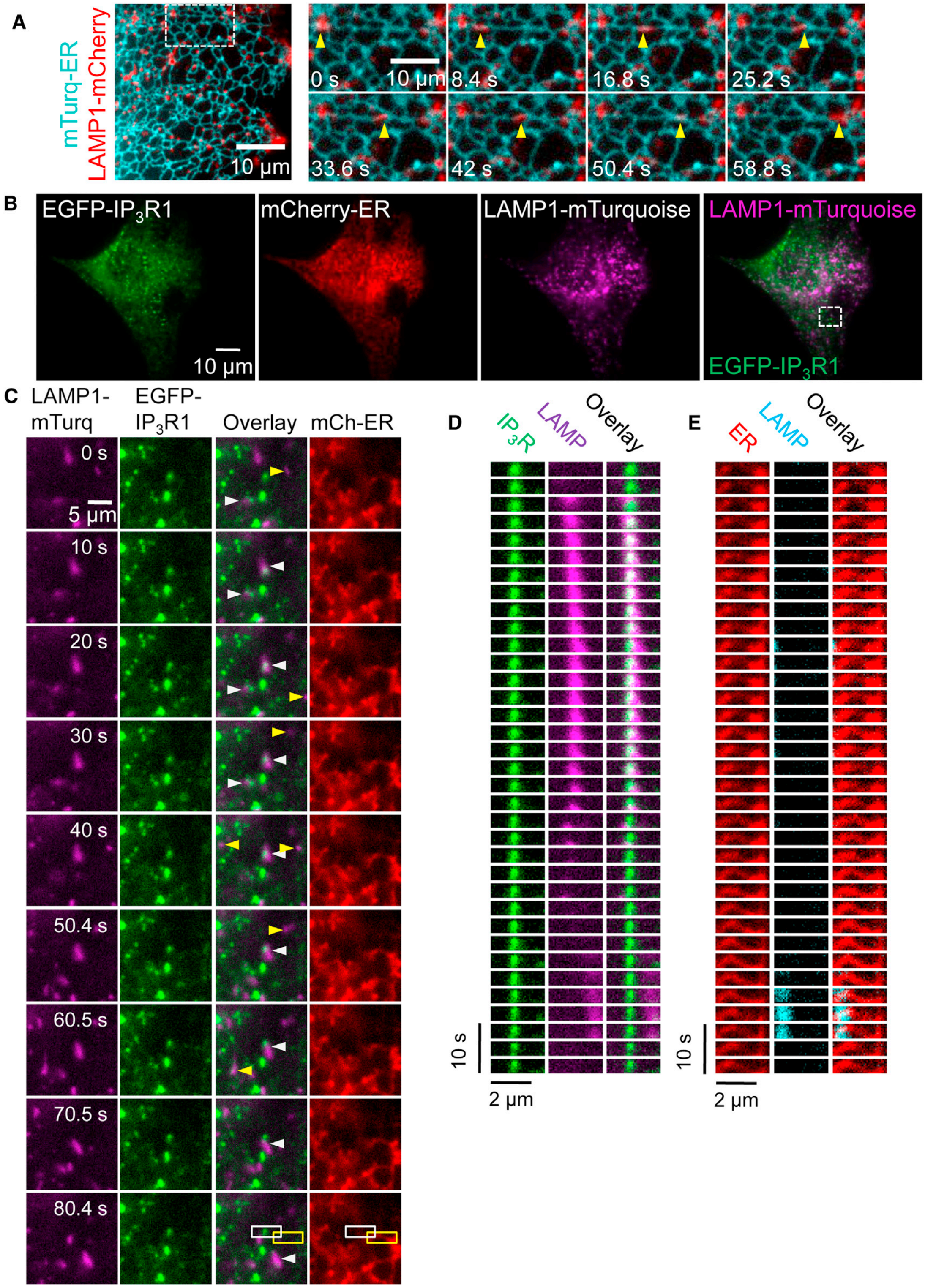

Figure 4. IP$_3$Rs Associate with ER-Lysosome Contacts

(A) TIRFM images of HeLa cell expressing mTurquoise-ER and LAMP1-mCherry showing that most lysosomes associate with ER and maintain contact as they move (yellow arrows). Images are shown at 8.4-s intervals (see Video S2).

(B) TIRFM images of EGFP-IP$_3$R1-HeLa cell expressing markers for lysosomes (mTurquoise-LAMP1) and ER (mCherry-ER). Merged image shows overlay of EGFP-IP$_3$R1 and lysosomes.

(legend continued on next page)

between lysosomes and IP_3Rs; mTurquoise-LAMP1 and mCherry-ER were used to identify lysosomes and ER using TIRFM (Figure 4B; Videos S4, S5, S6, S7, and S8). Within ER membranes, native IP_3Rs form small clusters, or puncta, that include an average of eight tetrameric IP_3Rs (Thillaiappan et al., 2017). Most lysosomes were mobile, but they often paused, sometimes for tens of seconds, at IP_3R puncta (Figure 4C). This behavior is best illustrated as time series, or kymograms, showing the distribution of the ER, IP_3R puncta, and lysosomes (Figures 4D, 4E, and S2–S4). We observed examples of lysosomes dwelling at immobile IP_3R puncta, but pausing only briefly at immediately adjacent ER (Figures 4D, 4E, and S2D; Video S4). Some lysosomes remained with an IP_3R punctum for tens of seconds before rapidly crossing intervening ER, and then dwelling at another immobile IP_3R punctum (Figures S2A and S2D; Videos S4 and S5). In other cases, an IP_3R punctum and a lysosome collided, and the pair stayed together as they moved or parked (Figures S2B and S2C; Video S6). We also observed two lysosomes associated with the same IP_3R punctum before one lysosome departed to associate with a different IP_3R punctum (Figure S3A). Finally, we observed examples of several lysosomes visiting the same IP_3R punctum at different times (Figure S3B; Video S7), and of a mobile IP_3R punctum pausing when it collided with an almost immobile lysosome (Figure S4; Video S8).

Because it was difficult to quantify the dynamic interactions between ER/IP_3Rs and lysosomes, we used *in situ* proximity ligation assays (PLA) to report whether an ER (VAP-A) and a lysosomal protein (Rab7) were located within ~40 nm of each other (Figure 5A). PLA detected many spots in HEK cells, indicative of VAP-A/Rab7 proximity, but not when either primary antibody was omitted, confirming the specificity of the PLA (Figures 5A–5C). We obtained similar results in EGFP-IP_3R1-HeLa cells (Figure 5D), where PLA reported the proximity of both VAP-A and EGFP-IP_3R to both Rab7 and another lysosomal protein (LAMP1) (Figures 5E and 5F). Furthermore, EGFP-IP_3R puncta and the PLA spots indicative of VAP-A/LAMP1 proximity were significantly colocalized (Manders's split coefficient, 0.70 ± 0.21, n = 18 cells, Costes's p value, 100%) (Figure 5D). Hence the MCSs between ER and lysosomes (revealed by the proximity of VAP-A to Rab7 or LAMP1) are populated by IP_3R puncta. Collectively, our results suggest that lysosomes preferentially associate with regions of ER where there are IP_3R puncta (Figures 4, 5, and S2–S4).

We considered whether IP_3Rs might contribute to formation of ER-lysosome MCSs, but two lines of evidence suggest this is unlikely. First, in HEK cells with and without IP_3Rs the association of lysosomes with ER was indistinguishable whether determined by PLA (Figures 5B and 5C) or their dynamic interactions (Figures 6A–6C; Videos S9 and S10). Second, we reported previously that the $[Ca^{2+}]_c$ increase evoked by inhibiting SERCA was exaggerated by bafilomycin A_1. We speculated that these signals might be due to Ca^{2+} leaking from the ER through translocons, IP_3Rs, or other unidentified channels (López Sanjurjo et al., 2013). However, bafilomycin A_1 potentiated the increase in $[Ca^{2+}]_c$ evoked by addition of thapsigargin in Ca^{2+}-free HBS to a similar extent in HEK cells with and without IP_3Rs (Figures 6D–6F). Similar results were obtained with HAP1 cells in which all endogenous IP_3R genes were disrupted (Figure S5). These results indicate that IP_3Rs mediate transfer of Ca^{2+} from ER to lysosomes in cells stimulated with CCh, but additional unidentified Ca^{2+} leak channels in the ER can deliver Ca^{2+} to lysosomes in unstimulated cells. We conclude that IP_3Rs associate with stable ER-lysosome MCSs, but they are not required for their assembly.

MCSs between ER and lysosomes are probably stabilized by tether proteins that may include VAP or protrudin (in ER), and STARD3, ORP1L, Rab7, TPC2, or phosphatidylinositol 3-phosphate (in lysosomes) (Kilpatrick et al., 2017; Phillips and Voeltz, 2016; Raiborg et al., 2015; Rocha et al., 2009). We manipulated ORP1L, which facilitates ER-lysosome contacts when cholesterol levels are low (Rocha et al., 2009), to assess whether it contributes to assembling the MCS where the Ca^{2+} exchanges occur. Expression of either ORP1L or forms expected to stabilize (ΔORD) or destabilize (ΔORDPHDPHD) ER-lysosome MCSs (Figure S6A) did not significantly affect potentiation of CCh-evoked Ca^{2+} signals by CcA (Figures S6B and S6C). However, expression of each ORP1L protein reduced the amplitude of the CCh-evoked Ca^{2+} signal (Figure S6B), but because this effect had no clear relationship to the expected effects of ORP1L proteins on MCS, we have not explored it further.

A Sustained Increase in Lysosomal pH Disrupts Lysosome Distribution and Ca^{2+} Handling

The Ca^{2+} uptake mechanism in mammalian lysosomes is unknown. It has been suggested to involve a low-affinity Ca^{2+}-H^+ exchanger (CAX), but there is no known CAX in mammalian cells (Melchionda et al., 2016; Morgan et al., 2011).

We used the ratiometric pH indicator, dextran-conjugated fluorescein, to measure lysosomal pH (Canton and Grinstein, 2015; Johnson et al., 2016). CcA (1 μM) caused a slow increase in lysosomal pH that reached a stable value after about 40 min (Figures 7A and 7B). Similar results were obtained when LysoTracker Red was used to report lysosomal pH (Figure 7C). In parallel analyses, we determined the effects of CcA on the Ca^{2+} signals evoked by CCh. As expected, prolonged incubation with CcA exaggerated the CCh-evoked increase in $[Ca^{2+}]_c$ (Figure 7D), consistent with normal lysosomes sequestering Ca^{2+} released through IP_3Rs (Figure 1I). However, the effect of CcA on CCh-evoked Ca^{2+} signals was much slower to develop than its effect on lysosomal pH. There was no evident effect of CcA on Ca^{2+} signals within 40 min, and a statistically significant potentiation of CCh-evoked Ca^{2+} signals required a 60-min

(C) Time-lapse TIRFM images (10-s intervals from Video S4) of boxed region in (B) show distribution of EGFP-IP_3R1, lysosomes, and ER. Arrows show examples of mobile lysosomes (yellow) and those that remain immobile for sustained periods (white). Immobile lysosomes coincide with IP_3R puncta. Mobile IP_3R puncta are not visible in these images because of the long capture intervals (3.3 s).
(D and E) Kymograms (3.3-s intervals) of boxed regions in (C) show a lysosome that is stationary for a prolonged period adjacent to an EGFP-IP_3R1 punctum (D, from white box in C), whereas another lysosome pauses only briefly at adjacent ER (E, from yellow box in C).
See also Figures S2, S3, and S6, and Videos S2, S3, S4, S5, S6, S7, and S8.

OPEN
ACCESS
CellPress

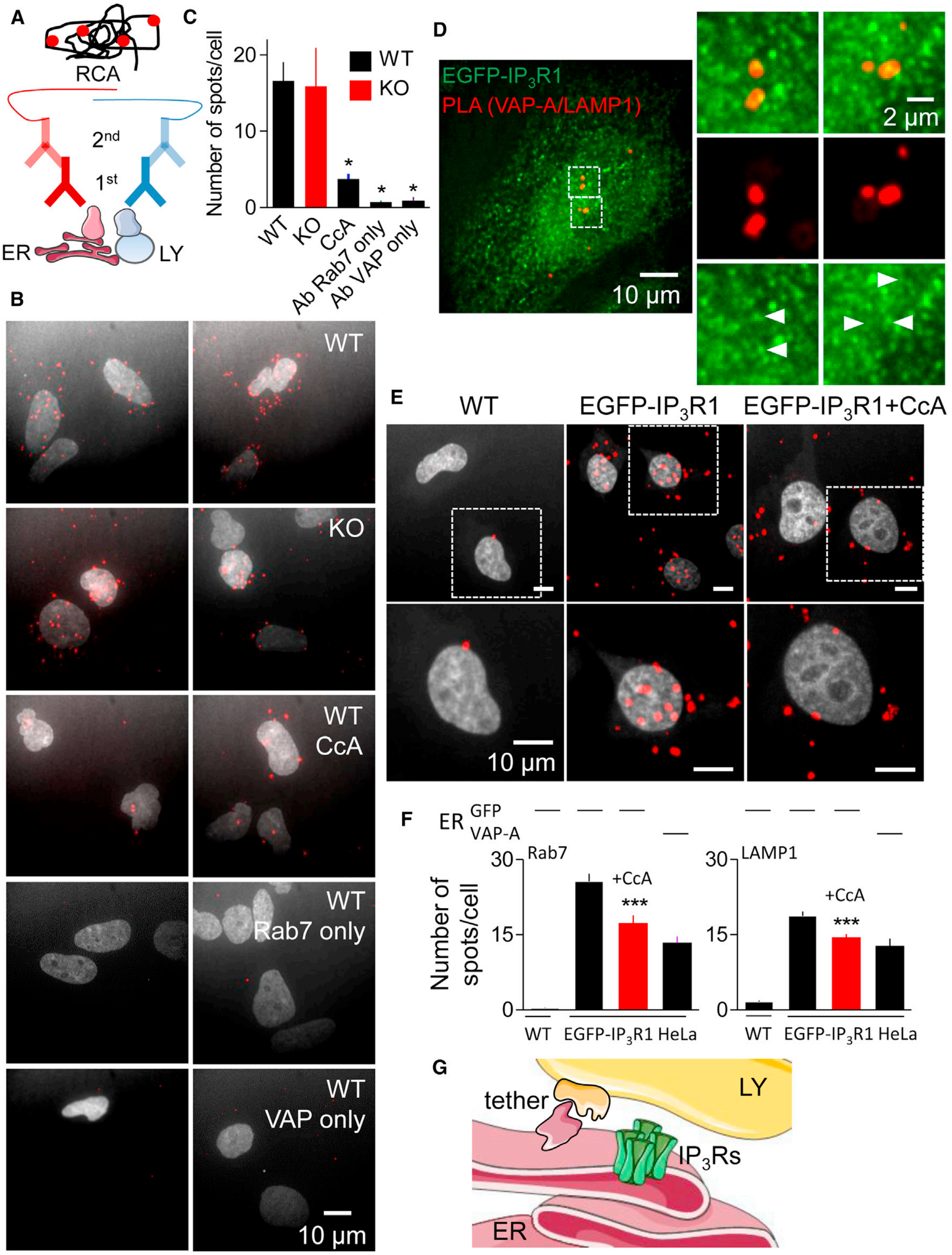

Figure 5. PLA Analyses Show IP$_3$Rs at ER-Lysosome MCSs

(A) PLA uses primary antibodies that recognize proteins in ER or lysosome (LY) membranes. Complementary oligonucleotides conjugated to secondary antibodies hybridize only if they are close to each other. Ligation of the hybridized strands then allows rolling circle amplification (RCA) of the oligonucleotide and incorporation of the red fluorescent probe.

(B) Images of HEK cells with (WT) and without (KO) IP$_3$Rs from PLA analyses of VAMP-associated protein A (VAP-A) proximity to Rab7. Confocal maximum intensity Z-projections show PLA spots (red) and nuclei (gray). Effects of CcA (1 μM, 1 hr) and omission of either primary antibody (Ab) are shown.

(legend continued on next page)

incubation with CcA (Figure 7D). These results show that Ca^{2+} sequestration by lysosomes persists after dissipation of the H^+ gradient, suggesting that compromised sequestration of the Ca^{2+} released through IP_3Rs may be a secondary consequence of the increase in lysosomal pH.

Lysosomes fuse when their pH increases (Cao et al., 2015) and the least acidic lysosomes are more peripherally located (Johnson et al., 2016). We therefore considered whether the delayed effects of CcA on CCh-evoked Ca^{2+} signals might be caused by redistribution of lysosomes. In most, although not all, HEK cells, prolonged treatment with CcA caused lysosomes to clearly redistribute from perinuclear to peripheral regions (Figures 7E and S7A); the redistribution was evident, but less striking, in the remaining cells. The same treatment caused a small, but statistically insignificant, decrease in the number of small lysosomes and a significant increase in the number of large ones (Figures 7F and S7B). These changes, suggesting fusion or ineffective fission of lysosomes, are similar to those evoked by knockdown of the core subunit of the V-ATPase (Figure 1F). Hence, prolonged dissipation of the lysosomal H^+ gradient causes lysosomes to enlarge and accumulate peripherally, and might thereby disrupt their interactions with IP_3Rs and ER. Quantification of the distance between each lysosome and the nearest IP_3R punctum confirmed that treatment with CcA increased the separation of lysosomes from native IP_3Rs (Figures 7G and 7H). PLA analyses independently verified that ER-lysosome MCSs, determined by the proximity of Rab7 to VAP-A or EGFP-IP_3R1, were disrupted by CcA (Figures 5B, 5C, 5E, and 5F). Peripheral, less acidic lysosomes express less Rab7 (Johnson et al., 2016), which could perturb our PLA analyses with CcA. We therefore reassessed the effects of CcA using PLA with a LAMP1 antibody to identify lysosomes. The results confirm that treatment with CcA reduces the number of ER-lysosome contacts (Figure 5F).

DISCUSSION

Selective Delivery of Ca^{2+} to Lysosomes from ER Ca^{2+} Channels

Dissipating the lysosomal pH gradient using inhibitors of the V-ATPase (López Sanjurjo et al., 2013, 2014) or knockdown of one of its core subunits exaggerates the cytosolic Ca^{2+} signals evoked by IP_3Rs, but not those evoked by SOCE (Figures 1 and S1). The effect, which is due to attenuated Ca^{2+} removal from cytosol rather than enhanced release from ER (López Sanjurjo et al., 2013), indicates that lysosomes selectively sequester Ca^{2+} released from the ER (Figure 1I). Others have used a Ca^{2+} sensor targeted to lysosomal membranes (LAMP1-yellow cameleon3.6) to detect Ca^{2+} signals evoked by histamine in HeLa cells (McCue et al., 2013), but this sensor with its high affinity for Ca^{2+} (K_D^{Ca} = 250 nM) (Nagai et al., 2004) cannot distinguish local from global $[Ca^{2+}]_c$ increases. To detect local increases in $[Ca^{2+}]_c$, we targeted a low-affinity sensor to lysosome membranes (Ly-GG, K_D^{Ca} = 1.2 μM) and tracked the responses of individual lysosomes (Video S1). Our results show that IP_3Rs, but not SOCE, deliver Ca^{2+} to about 60% of lysosomes (Figure 2). A similar situation prevails for mitochondria, where release of Ca^{2+} from IP_3Rs adjacent to mitochondria generates a high local $[Ca^{2+}]$, sufficient to allow Ca^{2+} uptake through the low-affinity mitochondrial uniporter complex (MCU) (Rizzuto et al., 2012). For mitochondria, IP_3R3 may, at least in some cells, selectively deliver Ca^{2+} to mitochondria (Cárdenas et al., 2010; Giorgi et al., 2018; Mendes et al., 2005). By contrast, our results show that all three IP_3R subtypes can deliver Ca^{2+} to lysosomes (Figure 3). We suggest that for both lysosomes and mitochondria, the ER through its SERCA and IP_3Rs provides a route through which Ca^{2+} can be accumulated from the low $[Ca^{2+}]_c$ of resting cells by a high-affinity uptake system (SERCA) and then delivered locally through large-conductance channels (IP_3Rs and perhaps others) at a high local concentration to a low-affinity uptake system (MCU or lysosomes) (Figure 7I). The ER, with its high-affinity SERCA and large-conductance Ca^{2+} channels, behaves like a compressor or piston linking a low $[Ca^{2+}]_c$ to the low-affinity uptake systems of organelles (Figure 7I).

IP_3Rs provide a link between extracellular stimuli and delivery of Ca^{2+} to lysosomes (Figures 1, 2, 3, and S1), but additional unidentified ER Ca^{2+} channels can deliver Ca^{2+} to lysosomes in unstimulated cells (Figures 6D–6F and S5). We propose, in keeping with a recent report (Garrity et al., 2016), that microdomains of high local $[Ca^{2+}]$ presented to lysosomes by IP_3Rs may facilitate lysosomal Ca^{2+} uptake (Figure 7I), and thereby link signaling through PLC to lysosome behavior.

IP_3R Clusters at ER-Lysosome Contacts Facilitate Ca^{2+} Transfer

MCS, where membranes of the ER and another organelle are held in close apposition by scaffold proteins, facilitate exchange of materials, including Ca^{2+}, between organelles (Figure 7I) (Phillips and Voeltz, 2016). Within the endosomal pathway, MCSs with the ER become more abundant as endosomes mature and acidify, such that most late endosomes form MCSs with the ER (Friedman et al., 2013) (Figure 4A; Videos S2 and S3). Several integral membrane components of the ER (e.g., VAP,

(C) Summary results (mean ± SD, n = 15–25 cells from three experiments [two experiments for single-antibody controls]) show number of PLA spots/cell. *$p < 0.05$, one-way ANOVA with Dunnett's test, relative to WT control.
(D) Confocal section of PLA in EGFP-IP_3R1 HeLa cells shows VAP-A proximity to LAMP1 (red) and endogenously tagged IP_3R1 (green). Boxed areas are shown enlarged to illustrate the coincidence of EGFP-IP_3R puncta with PLA spots (ER-lysosome MCS) (Manders's split coefficient, 0.70 ± 0.21, n = 18 cells).
(E) Confocal maximum intensity Z-projections of PLA in EGFP-IP_3R1 HeLa cells using antibodies to GFP and LAMP1 to show their proximity (red spots). Nucleus is shown in gray. Effects of CcA (1 μM, 1 hr) and of performing the same PLA in wild-type (WT) HeLa cells without EGFP-IP_3R1 are also shown.
(F) Summary PLA results using Rab7 (left panel) or LAMP1 (right) to identify lysosomes and either GFP (from EGFP-IP_3R1) or VAP-A to identify ER (shown by bars above the histograms). Mean ± SD, n = 25–97 cells from 3–5 experiments. ***$p < 0.001$, Student's t test for CcA-treated relative to matched control EGFP-IP_3R1-HeLa cell.
(G) Most lysosomes (LY) are closely associated, aided by tethers, with ER at MCS. Small clusters of IP_3Rs associate with these MCS, but IP_3Rs are not required for their assembly.
WT, wild-type. See also Figures S2 and S6 and Videos S2, S3, S4, S5, S6, S7, and S8.

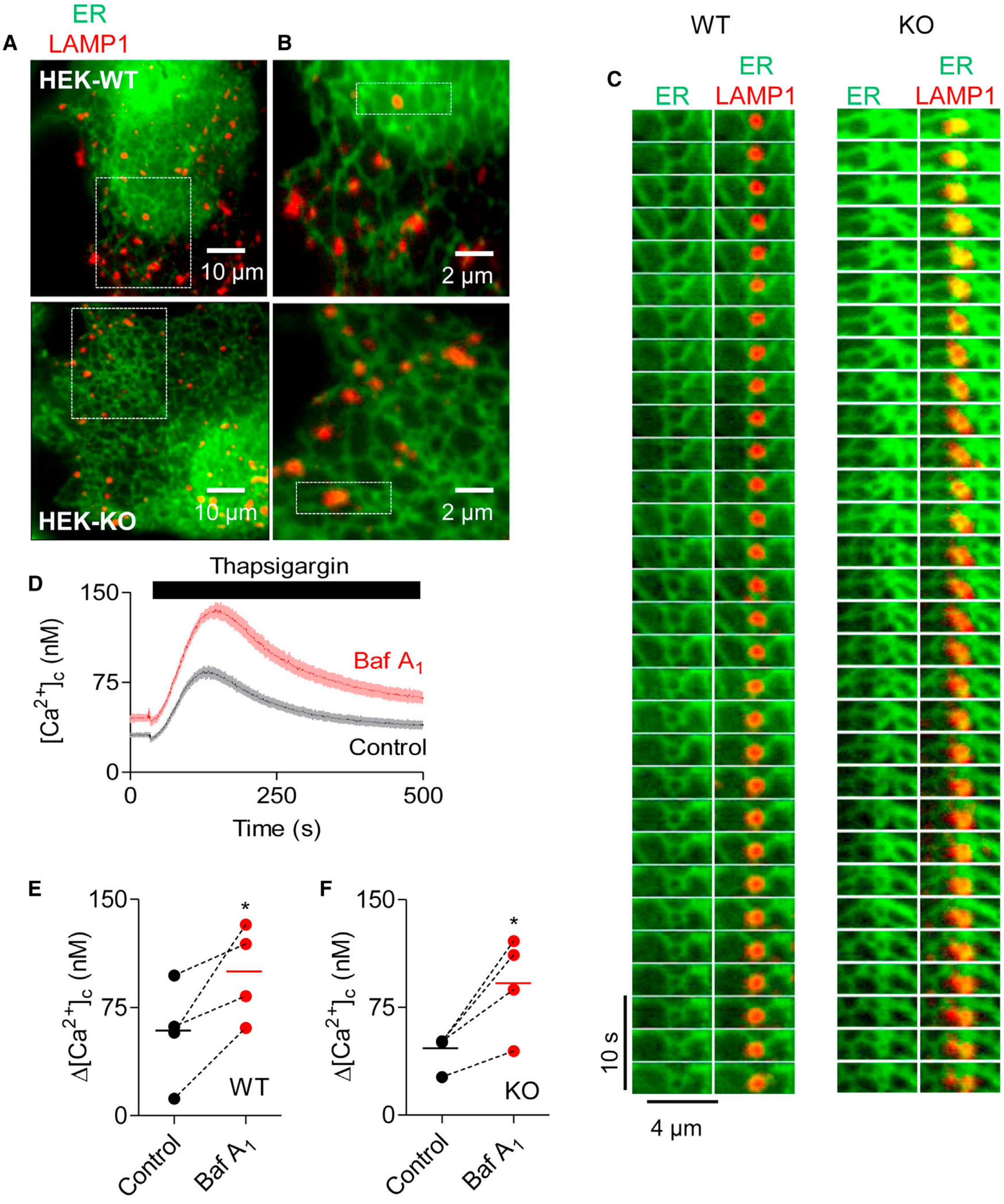

Figure 6. IP_3Rs Are Not Required for ER-Lysosome Contacts

(A) TIRFM images of HEK cells with (WT) and without IP_3Rs (KO), expressing LAMP1-mCherry and EGFP-ER.

(B) Enlargements of boxed region in (A) show associations of lysosomes and ER.

(C) Time series (3-s intervals) of boxed regions in (B) show dynamics of ER-lysosome interactions (from Videos S9 and S10).

(D) HEK cells were treated with bafilomycin A_1 (Baf A_1, 1 μM, 1 hr) in HBS before addition of BAPTA (2.5 mM) and then thapsigargin (1 μM). Mean ± SD from three wells in one experiment.

(E and F) Summary results show peak thapsigargin-evoked Ca^{2+} release in WT (E) and KO cells (F) as paired values (each with three determinations) and mean (n = 4, line). *$p < 0.05$, paired Student's t test.

See also Figures S5 and S6 and Videos S9 and S10.

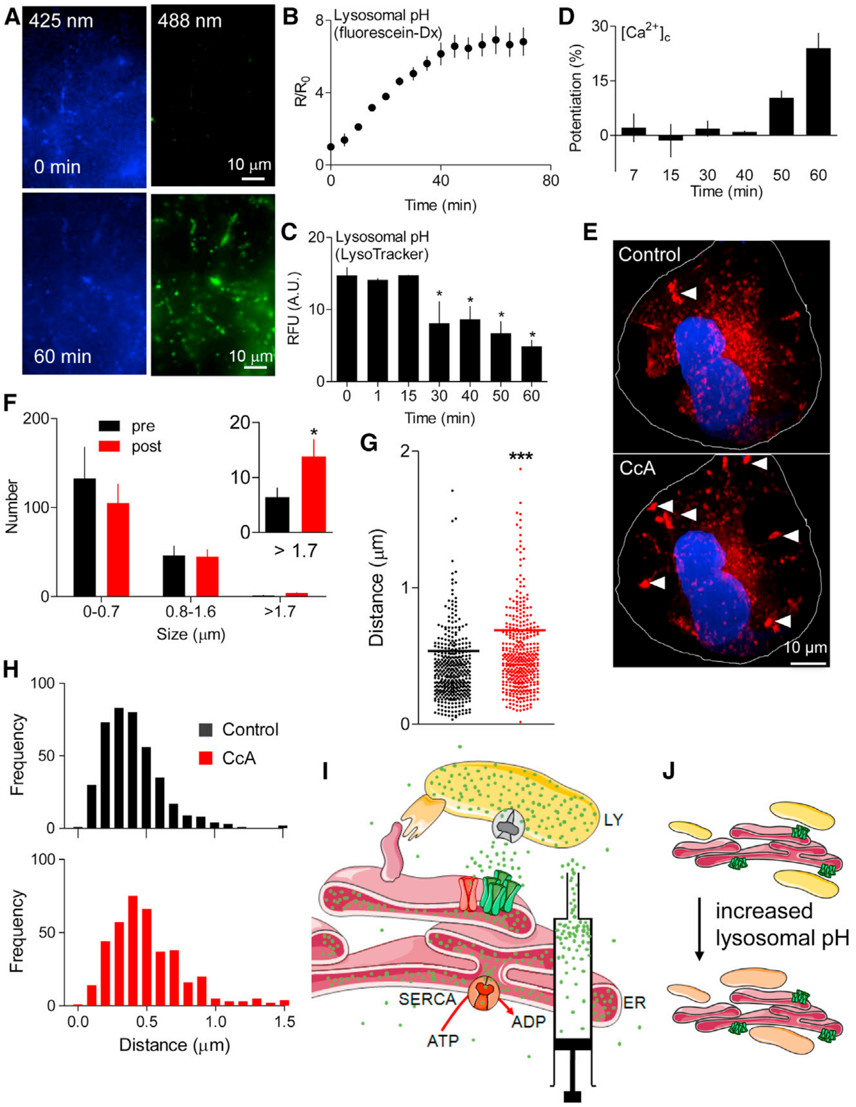

Figure 7. Increasing Lysosomal pH Slowly Redistributes Lysosomes and Attenuates Ca^{2+} Handling

(A) Lysosomes of HEK cells were loaded with fluorescein-dextran (pK_a = 6.4). Wide-field images show fluorescence recorded at pH-sensitive (λ_{ex} = 488 nm) and -insensitive (λ_{ex} = 425 nm) wavelengths before (0 min) and after CcA (1 μM, 1 hr). Images are typical of three experiments.

(B) Summary results (mean ± SEM, n = 3) show time course of lysosomal pH changes after CcA as fluorescence ratios ($R = F_{488}/F_{425}$), which increase as pH increases. R_0 is R determined before CcA.

(C) Effects of CcA (1 μM) using LysoTracker red (50 nM, 1 hr) fluorescence, which declines as lysosomal pH increases. Mean ± SEM, n = 4–10. *$p < 0.05$, one-way ANOVA with Dunnett's post hoc test, relative to t = 0.

(D) Parallel analysis of CcA (1 μM) effects on peak increase in $[Ca^{2+}]_c$ evoked by CCh (1 mM) in Ca^{2+}-free HBS. Results (mean ± SEM, n = 4–9, with three determinations) show increase in peak Ca^{2+} signal in the presence of CcA relative to that in its absence, as percentage of response evoked by CCh alone.

(E) Confocal z stack of HEK cells expressing LAMP1-mCherry before and after CcA (1 μM, 1 hr). Nuclei stained with NucBlue. The appearance of larger lysosomes in the cell periphery (white arrows) following CcA (1 μM, 1 hr) was clearly observed in four of six cells.

(F) Effect of CcA (1 μM, 1 hr) on distribution of lysosome sizes (reported as Feret diameter, see STAR Methods). Results are from 721 (control) and 617 lysosomes (CcA-treated) from 4 cells in 4 independent experiments. Inset shows enlargement of the largest size category. *$p < 0.05$, Student's t test. A similar analysis using lysosomes identified by an endocytosed fluorophore is shown in Figure S7.

(G and H) Effects of CcA (1 μM, 1 hr) on the distance between each lysosome and the nearest IP_3R punctum. Because these distances are reported as centroid-centroid separations, they can be smaller than the diffraction limit of the microscope. Results from four cells, with ~20 × 20 μm analyzed in each, show each measurement and mean (line) (G) and frequency distributions (H). ***$p < 0.001$, two-tailed Student's t test.

(I) Delivery of Ca^{2+} through IP_3Rs or unidentified "leak channels" into MCSs provides a low-affinity Ca^{2+} uptake system in lysosomes with the high local $[Ca^{2+}]$ required for its activity. The ER, with its high-affinity Ca^{2+} pump (SERCA), accumulates Ca^{2+} from the cytosol and delivers it at high local concentration to the surface of lysosomes through large-conductance IP_3Rs. ER behaves as an ATP-powered piston to concentrate Ca^{2+} around the lysosomal uptake system.

(J) Dissipating lysosomal pH gradient does not immediately prevent lysosomal Ca^{2+} uptake, but slowly disrupts junctions within which it occurs.

See also Figure S7.

protrudin) and endosomes (e.g., STARD3, ORP1L, Rab7, phosphatidylinositol 3-phosphate) are implicated in the formation of MCSs between the ER and late endosomes (Alpy et al., 2013; Eden, 2016; Hong et al., 2017; Phillips and Voeltz, 2016; Raiborg et al., 2015), and both Ca^{2+} signals within MCSs and functional TPC1 may be required for their maintenance (Kilpatrick et al., 2017). The components of lysosome-ER MCSs are less defined, although it has been suggested that TPC2 may fulfill a similar role here to that suggested for TPC1 at endosome-ER MCSs (Kilpatrick et al., 2017). Hence, although it is widely supposed that MCSs mediate exchange of, for example, Ca^{2+} and cholesterol, between ER and lysosomes, the composition of these MCSs is unclear. Our analyses suggest that ORP1L is unlikely to be an essential component of the MCSs wherein Ca^{2+} is delivered from the ER to lysosomes (Figure S6).

We confirmed that most lysosomes maintain contact with the ER, despite movements of both organelles (Figure 4A; Videos S2 and S3) (López Sanjurjo et al., 2013). The association reflects close apposition of the ER and lysosome membranes (Figure 5). The most persistent associations between lysosomes and the ER coincide with regions that are populated by clusters of native IP_3Rs, and the affiliation of IP_3Rs with lysosomes occurs at both moving and immotile contacts (Figures 4, 5D–5F, and S2–S4; Videos S4, S5, S6, S7, and S8). Although IP_3Rs populate the

ER-lysosome MCSs, our results suggest they do not contribute to assembly of the MCSs. Sequestration by lysosomes of Ca^{2+} released from the ER through leak channels is similar in cells with and without IP_3Rs (Figures 6D–6F and S5); the persistent association of lysosomes with the ER, which in normal cells often coincides with IP_3R puncta (Figures 4C–4E and S2–S4; Videos S4, S5, S6, S7, and S8), is similar in cells with and without IP_3Rs (Figures 6A–6C and S5; Videos S9 and S10), and the proximity of ER (VAP-A) and a lysosome protein (Rab7) determined by PLA is indistinguishable in cells with and without IP_3Rs (Figures 5B and 5C). We conclude that IP_3R puncta associate with stable ER-lysosome MCSs, but IP_3Rs are not required for assembly of these contacts.

How Do Lysosomes Accumulate Ca^{2+}?

Ca^{2+} uptake by mammalian lysosomes has been suggested to involve a low-affinity CAX, consistent with evidence that dissipating the lysosomal H^+ gradient increases $[Ca^{2+}]_c$ (Lloyd-Evans et al., 2008) and decreases lysosomal free $[Ca^{2+}]$ (Christensen et al., 2002), with Ca^{2+} uptake causing an increase in lysosomal pH (Hilden and Madias, 1989; López Sanjurjo et al., 2013; Morgan and Galione, 2007) and with heterologous expression of *Xenopus* CAX in mammalian cells attenuating CCh-evoked Ca^{2+} signals (Melchionda et al., 2016). However, there is no known CAX in mammalian cells (Melchionda et al., 2016; Morgan et al., 2011).

Our results show that, although dissipating the lysosomal pH gradient inhibits Ca^{2+} uptake by lysosomes, there is a temporal mismatch between the effects of CcA on lysosomal pH and attenuated Ca^{2+} uptake, with the latter developing much more slowly (Figures 7A–7D). A recent study, in which a low-affinity Ca^{2+} sensor tethered to TRPML1 was used to report refilling of lysosomes, likewise concluded that treatments with bafilomycin A_1 or CcA that abolished the lysosomal pH gradient did not prevent refilling of lysosomes with Ca^{2+} (Garrity et al., 2016). Hence, and consistent with the apparent absence of CAX from mammalian cells, lysosomes can, at least acutely, accumulate Ca^{2+} in the absence of a pH gradient. Why then does sustained inhibition of the V-ATPase prevent lysosomes from sequestering Ca^{2+} released from the ER (Figures 1, 3, and S1)?

We suggest that this inhibition results from disruption of the contacts between ER and lysosomes. This is consistent with evidence that vacuolin, which fuses lysosomes (Huynh and Andrews, 2005), also prevents lysosomes from sequestering Ca^{2+} released from the ER (López Sanjurjo et al., 2013). Sustained inhibition of the V-ATPase causes the fraction of the cell occupied by lysosomes to decrease by $\sim$20% (Abu-Remaileh et al., 2017), enlargement of lysosomes (Figures 1F, 7F, and S7), redistribution of lysosomes from perinuclear to peripheral regions (Figures 7E and S7), and disruption of both ER-lysosome MCSs (Figures 5B, 5C, 5E, and 5F) and the association of IP_3Rs with lysosomes (Figures 5D–5F, 7G, and 7H). These observations are consistent with results showing that lysosomal pH and/or local Ca^{2+} signals regulate fusion and/or fission of lysosomes (Baars et al., 2007; Cao et al., 2017; Coen et al., 2012; Kilpatrick et al., 2017; Ruas et al., 2010) and their subcellular distribution (Johnson et al., 2016). We suggest that the inability of lysosomes to sequester Ca^{2+} released from ER after sustained inhibition of the V-ATPase results from a disruption of the MCSs where Ca^{2+} transfer occurs between lysosomes and ER (Figure 7J). Our evidence that CcA increases the separation of lysosomes and native IP_3Rs supports this suggestion (Figures 5D–5F, 7G, and 7H). We have not determined whether all Ca^{2+} uptake by lysosomes occurs at these MCSs, but others have suggested that IP_3R-mediated transfer from the ER is the only means by which lysosomes sequester Ca^{2+} (Garrity et al., 2016). We suggest that MCSs between ER and lysosomes are populated by clusters of IP_3Rs, poised to deliver Ca^{2+} at high local concentrations to a low-affinity, but as yet unidentified, lysosomal Ca^{2+} uptake mechanism (Figures 7I). Because the distribution of lysosomes and their association with ER are regulated by amino acids (Hong et al., 2017), it is likely that dynamic regulation of ER-lysosome MCSs also regulates lysosomal Ca^{2+} uptake.

STAR★METHODS

Detailed methods are provided in the online version of this paper and include the following:

- KEY RESOURCES TABLE
- CONTACT FOR REAGENT AND RESOURCE SHARING
- EXPERIMENTAL MODEL AND SUBJECT DETAILS
  - Cell Culture and Transfection
- METHOD DETAILS
  - Plasmids
  - Measurements of $[Ca^{2+}]_c$ in Cell Populations
  - Fluorescence Microscopy
  - Measurement of Near-Lysosome Ca^{2+} Signals
  - Photolysis of Caged-IP_3
  - Measurement of Lysosomal pH
  - Measurement of Lysosome Size
  - Quantitative PCR
  - Proximity Ligation Assays
  - Western Blots
- QUANTIFICATION AND STATISTICAL ANALYSIS

SUPPLEMENTAL INFORMATION

Supplemental Information includes seven figures, one table, and ten videos and can be found with this article online at https://doi.org/10.1016/j.celrep.2018.11.064.

ACKNOWLEDGMENTS

This work was supported by the Wellcome Trust (grant 101844 to C.W.T.), the Biotechnology and Biological Sciences Research Council UK (grant BB/P005330/1 to C.W.T.), and research studentships from the Cambridge Commonwealth, European, and International Trust (to P.A.) and Biotechnology and Biological Sciences Research Council UK Doctoral Training Programme (to S.M.). We thank Marharyta Kamarova for help with preliminary experiments, David Yule (University of Rochester, USA) for providing HEK cells lacking IP_3Rs, and Jacques Neefjes (Leiden University Medical Center, the Netherlands) for ORP1L plasmids.

AUTHOR CONTRIBUTIONS

P.A. and N.B.T. performed most experiments. S.M. characterized HAP1 cells. D.L.P. contributed to development of G-GECO sensors. C.W.T. supervised the

project and contributed to data analysis. All authors contributed to writing and reviewing the paper.

DECLARATION OF INTERESTS

The authors declare no competing interests.

Received: April 5, 2018
Revised: July 30, 2018
Accepted: November 15, 2018
Published: December 11, 2018

REFERENCES

Abu-Remaileh, M., Wyant, G.A., Kim, C., Laqtom, N.N., Abbasi, M., Chan, S.H., Freinkman, E., and Sabatini, D.M. (2017). Lysosomal metabolomics reveals V-ATPase- and mTOR-dependent regulation of amino acid efflux from lysosomes. Science *358*, 807–813.

Alpy, F., Rousseau, A., Schwab, Y., Legueux, F., Stoll, I., Wendling, C., Spiegelhalter, C., Kessler, P., Mathelin, C., Rio, M.C., et al. (2013). STARD3 or STARD3NL and VAP form a novel molecular tether between late endosomes and the ER. J. Cell Sci. *126*, 5500–5512.

Alzayady, K.J., Wang, L., Chandrasekhar, R., Wagner, L.E., 2nd, Van Petegem, F., and Yule, D.I. (2016). Defining the stoichiometry of inositol 1,4,5-trisphosphate binding required to initiate Ca^{2+} release. Sci. Signal. *9*, ra35.

Baars, T.L., Petri, S., Peters, C., and Mayer, A. (2007). Role of the V-ATPase in regulation of the vacuolar fission-fusion equilibrium. Mol. Biol. Cell *18*, 3873–3882.

Brailoiu, E., Churamani, D., Cai, X., Schrlau, M.G., Brailoiu, G.C., Gao, X., Hooper, R., Boulware, M.J., Dun, N.J., Marchant, J.S., and Patel, S. (2009). Essential requirement for two-pore channel 1 in NAADP-mediated calcium signaling. J. Cell Biol. *186*, 201–209.

Canton, J., and Grinstein, S. (2015). Measuring lysosomal pH by fluorescence microscopy. Methods Cell Biol. *126*, 85–99.

Cao, Q., Zhong, X.Z., Zou, Y., Murrell-Lagnado, R., Zhu, M.X., and Dong, X.P. (2015). Calcium release through P2X4 activates calmodulin to promote endo-lysosomal membrane fusion. J. Cell Biol. *209*, 879–894.

Cao, Q., Yang, Y., Zhong, X.Z., and Dong, X.P. (2017). The lysosomal Ca^{2+} release channel TRPML1 regulates lysosome size by activating calmodulin. J. Biol. Chem. *292*, 8424–8435.

Cárdenas, C., Miller, R.A., Smith, I., Bui, T., Molgó, J., Müller, M., Vais, H., Cheung, K.H., Yang, J., Parker, I., et al. (2010). Essential regulation of cell bioenergetics by constitutive $InsP_3$ receptor Ca^{2+} transfer to mitochondria. Cell *142*, 270–283.

Christensen, K.A., Myers, J.T., and Swanson, J.A. (2002). pH-dependent regulation of lysosomal calcium in macrophages. J. Cell Sci. *115*, 599–607.

Coen, K., Flannagan, R.S., Baron, S., Carraro-Lacroix, L.R., Wang, D., Vermeire, W., Michiels, C., Munck, S., Baert, V., Sugita, S., et al. (2012). Lysosomal calcium homeostasis defects, not proton pump defects, cause endo-lysosomal dysfunction in PSEN-deficient cells. J. Cell Biol. *198*, 23–35.

Costes, S.V., Daelemans, D., Cho, E.H., Dobbin, Z., Pavlakis, G., and Lockett, S. (2004). Automatic and quantitative measurement of protein-protein colocalization in live cells. Biophys. J. *86*, 3993–4003.

Courjaret, R., Dib, M., and Machaca, K. (2017). Store-operated Ca^{2+} entry in oocytes modulate the dynamics of IP_3-dependent Ca^{2+} release from oscillatory to tonic. J. Cell. Physiol. *232*, 1095–1103.

Dakin, K., and Li, W.H. (2007). Cell membrane permeable esters of D-*myo*-inositol 1,4,5-trisphosphate. Cell Calcium *42*, 291–301.

Dröse, S., Bindseil, K.U., Bowman, E.J., Siebers, A., Zeeck, A., and Altendorf, K. (1993). Inhibitory effect of modified bafilomycins and concanamycins on P- and V-type adenosinetriphosphatases. Biochemistry *32*, 3902–3906.

Eden, E.R. (2016). The formation and function of ER-endosome membrane contact sites. Biochim. Biophys. Acta *1861*, 874–879.

Ferraro, F., Kriston-Vizi, J., Metcalf, D.J., Martin-Martin, B., Freeman, J., Burden, J.J., Westmoreland, D., Dyer, C.E., Knight, A.E., Ketteler, R., and Cutler, D.F. (2014). A two-tier Golgi-based control of organelle size underpins the functional plasticity of endothelial cells. Dev. Cell *29*, 292–304.

Forgac, M. (2007). Vacuolar ATPases: rotary proton pumps in physiology and pathophysiology. Nat. Rev. Mol. Cell Biol. *8*, 917–929.

Foskett, J.K., White, C., Cheung, K.H., and Mak, D.O. (2007). Inositol trisphosphate receptor Ca^{2+} release channels. Physiol. Rev. *87*, 593–658.

Fredriksson, S., Gullberg, M., Jarvius, J., Olsson, C., Pietras, K., Gústafsdóttir, S.M., Ostman, A., and Landegren, U. (2002). Protein detection using proximity-dependent DNA ligation assays. Nat. Biotechnol. *20*, 473–477.

Friedman, J.R., Dibenedetto, J.R., West, M., Rowland, A.A., and Voeltz, G.K. (2013). Endoplasmic reticulum-endosome contact increases as endosomes traffic and mature. Mol. Biol. Cell *24*, 1030–1040.

Galione, A. (2015). A primer of NAADP-mediated Ca^{2+} signalling: from sea urchin eggs to mammalian cells. Cell Calcium *58*, 27–47.

Garrity, A.G., Wang, W., Collier, C.M., Levey, S.A., Gao, Q., and Xu, H. (2016). The endoplasmic reticulum, not the pH gradient, drives calcium refilling of lysosomes. Elife *5*, e15887.

Gilles, J.F., Dos Santos, M., Boudier, T., Bolte, S., and Heck, N. (2017). DiAna, an ImageJ tool for object-based 3D co-localization and distance analysis. Methods *115*, 55–64.

Giorgi, C., Danese, A., Missiroli, S., Patergnani, S., and Pinton, P. (2018). Calcium dynamics as a machine for decoding signals. Trends Cell Biol. *28*, 258–273.

Grossi, M., Morgunova, M., Cheung, S., Scholz, D., Conroy, E., Terrile, M., Panarella, A., Simpson, J.C., Gallagher, W.M., and O'Shea, D.F. (2016). Lysosome triggered near-infrared fluorescence imaging of cellular trafficking processes in real time. Nat. Commun. *7*, 10855.

Hilden, S.A., and Madias, N.E. (1989). H^+/Ca^{2+} exchange in rabbit renal cortical endosomes. J. Membr. Biol. *112*, 131–138.

Hong, Z., Pedersen, N.M., Wang, L., Torgersen, M.L., Stenmark, H., and Raiborg, C. (2017). PtdIns3P controls mTORC1 signaling through lysosomal positioning. J. Cell Biol. *216*, 4217–4233.

Huynh, C., and Andrews, N.W. (2005). The small chemical vacuolin-1 alters the morphology of lysosomes without inhibiting Ca^{2+}-regulated exocytosis. EMBO Rep. *6*, 843–847.

Johnson, D.E., Ostrowski, P., Jaumouillé, V., and Grinstein, S. (2016). The position of lysosomes within the cell determines their luminal pH. J. Cell Biol. *212*, 677–692.

Kilpatrick, B.S., Eden, E.R., Hockey, L.N., Yates, E., Futter, C.E., and Patel, S. (2017). An endosomal NAADP-sensitive two-pore Ca^{2+} channel regulates ER-endosome membrane contact sites to control growth factor signaling. Cell Rep. *18*, 1636–1645.

Koos, B., Andersson, L., Clausson, C.M., Grannas, K., Klaesson, A., Cane, G., and Söderberg, O. (2014). Analysis of protein interactions in situ by proximity ligation assays. Curr. Top. Microbiol. Immunol. *377*, 111–126.

Lloyd-Evans, E., Morgan, A.J., He, X., Smith, D.A., Elliot-Smith, E., Sillence, D.J., Churchill, G.C., Schuchman, E.H., Galione, A., and Platt, F.M. (2008). Niemann-Pick disease type C1 is a sphingosine storage disease that causes deregulation of lysosomal calcium. Nat. Med. *14*, 1247–1255.

López-Sanjurjo, C.I., Tovey, S.C., Prole, D.L., and Taylor, C.W. (2013). Lysosomes shape Ins(1,4,5)P_3-evoked Ca^{2+} signals by selectively sequestering Ca^{2+} released from the endoplasmic reticulum. J. Cell Sci. *126*, 289–300.

López Sanjurjo, C.I., Tovey, S.C., and Taylor, C.W. (2014). Rapid recycling of Ca^{2+} between IP_3-sensitive stores and lysosomes. PLoS ONE *9*, e111275.

Mangieri, L.R., Mader, B.J., Thomas, C.E., Taylor, C.A., Luker, A.M., Tse, T.E., Huisingh, C., and Shacka, J.J. (2014). ATP6V0C knockdown in neuroblastoma cells alters autophagy-lysosome pathway function and metabolism of proteins that accumulate in neurodegenerative disease. PLoS ONE *9*, e93257.

Mataragka, S., and Taylor, C.W. (2018). All three IP_3 receptor subtypes generate Ca^{2+} puffs, the universal building blocks of IP_3-evoked Ca^{2+} signals. J. Cell Sci. *131*, jcs220848.

McCue, H.V., Wardyn, J.D., Burgoyne, R.D., and Haynes, L.P. (2013). Generation and characterization of a lysosomally targeted, genetically encoded Ca^{2+}-sensor. Biochem. J. *449*, 449–457.

Medina, D.L., Di Paola, S., Peluso, I., Armani, A., De Stefani, D., Venditti, R., Montefusco, S., Scotto-Rosato, A., Prezioso, C., Forrester, A., et al. (2015). Lysosomal calcium signalling regulates autophagy through calcineurin and TFEB. Nat. Cell Biol. *17*, 288–299.

Meijering, E., Dzyubachyk, O., and Smal, I. (2012). Methods for cell and particle tracking. Methods Enzymol. *504*, 183–200.

Melchionda, M., Pittman, J.K., Mayor, R., and Patel, S. (2016). Ca^{2+}/H^+ exchange by acidic organelles regulates cell migration in vivo. J. Cell Biol. *212*, 803–813.

Mendes, C.C., Gomes, D.A., Thompson, M., Souto, N.C., Goes, T.S., Goes, A.M., Rodrigues, M.A., Gomez, M.V., Nathanson, M.H., and Leite, M.F. (2005). The type III inositol 1,4,5-trisphosphate receptor preferentially transmits apoptotic Ca^{2+} signals into mitochondria. J. Biol. Chem. *280*, 40892–40900.

Morgan, A.J., and Galione, A. (2007). NAADP induces pH changes in the lumen of acidic Ca^{2+} stores. Biochem. J. *402*, 301–310.

Morgan, A.J., Platt, F.M., Lloyd-Evans, E., and Galione, A. (2011). Molecular mechanisms of endolysosomal Ca^{2+} signalling in health and disease. Biochem. J. *439*, 349–374.

Morgan, A.J., Davis, L.C., Wagner, S.K., Lewis, A.M., Parrington, J., Churchill, G.C., and Galione, A. (2013). Bidirectional Ca^{2+} signaling occurs between the endoplasmic reticulum and acidic organelles. J. Cell Biol. *200*, 789–805.

Nagai, T., Yamada, S., Tominaga, T., Ichikawa, M., and Miyawaki, A. (2004). Expanded dynamic range of fluorescent indicators for Ca^{2+} by circularly permuted yellow fluorescent proteins. Proc. Natl. Acad. Sci. USA *101*, 10554–10559.

Nelson, M.T., Cheng, H., Rubart, M., Santana, L.F., Bonev, A.D., Knot, H.J., and Lederer, W.J. (1995). Relaxation of arterial smooth muscle by calcium sparks. Science *270*, 633–637.

Patel, S., Marchant, J.S., and Brailoiu, E. (2010). Two-pore channels: regulation by NAADP and customized roles in triggering calcium signals. Cell Calcium *47*, 480–490.

Phillips, M.J., and Voeltz, G.K. (2016). Structure and function of ER membrane contact sites with other organelles. Nat. Rev. Mol. Cell Biol. *17*, 69–82.

Prakriya, M., and Lewis, R.S. (2015). Store-operated calcium channels. Physiol. Rev. *95*, 1383–1436.

Raiborg, C., Wenzel, E.M., Pedersen, N.M., Olsvik, H., Schink, K.O., Schultz, S.W., Vietri, M., Nisi, V., Bucci, C., Brech, A., et al. (2015). Repeated ER-endosome contacts promote endosome translocation and neurite outgrowth. Nature *520*, 234–238.

Ríos, E. (2018). Calcium-induced release of calcium in muscle: 50 years of work and the emerging consensus. J. Gen. Physiol. *150*, 521–537.

Rizzuto, R., De Stefani, D., Raffaello, A., and Mammucari, C. (2012). Mitochondria as sensors and regulators of calcium signalling. Nat. Rev. Mol. Cell Biol. *13*, 566–578.

Rocha, N., Kuijl, C., van der Kant, R., Janssen, L., Houben, D., Janssen, H., Zwart, W., and Neefjes, J. (2009). Cholesterol sensor ORP1L contacts the ER protein VAP to control Rab7-RILP-p150 Glued and late endosome positioning. J. Cell Biol. *185*, 1209–1225.

Ruas, M., Rietdorf, K., Arredouani, A., Davis, L.C., Lloyd-Evans, E., Koegel, H., Funnell, T.M., Morgan, A.J., Ward, J.A., Watanabe, K., et al. (2010). Purified TPC isoforms form NAADP receptors with distinct roles for Ca^{2+} signaling and endolysosomal trafficking. Curr. Biol. *20*, 703–709.

Schindelin, J., Arganda-Carreras, I., Frise, E., Kaynig, V., Longair, M., Pietzsch, T., Preibisch, S., Rueden, C., Saalfeld, S., Schmid, B., et al. (2012). Fiji: an open-source platform for biological-image analysis. Nat. Methods *9*, 676–682.

Taylor, C.W., and Tovey, S.C. (2010). IP_3 receptors: toward understanding their activation. Cold Spring Harb. Perspect. Biol. *2*, a004010.

Thillaiappan, N.B., Chavda, A.P., Tovey, S.C., Prole, D.L., and Taylor, C.W. (2017). Ca^{2+} signals initiate at immobile IP_3 receptors adjacent to ER-plasma membrane junctions. Nat. Commun. *8*, 1505.

Tovey, S.C., Sun, Y., and Taylor, C.W. (2006). Rapid functional assays of intracellular Ca^{2+} channels. Nat. Protoc. *1*, 259–263.

Tovey, S.C., Dedos, S.G., Taylor, E.J.A., Church, J.E., and Taylor, C.W. (2008). Selective coupling of type 6 adenylyl cyclase with type 2 IP_3 receptors mediates direct sensitization of IP_3 receptors by cAMP. J. Cell Biol. *183*, 297–311.

Zhao, Y., Araki, S., Wu, J., Teramoto, T., Chang, Y.F., Nakano, M., Abdelfattah, A.S., Fujiwara, M., Ishihara, T., Nagai, T., and Campbell, R.E. (2011). An expanded palette of genetically encoded Ca^{2+} indicators. Science *333*, 1888–1891.

STAR★METHODS

KEY RESOURCES TABLE

REAGENT OR RESOURCE	SOURCE	IDENTIFIER
Antibodies		
Donkey anti-rabbit IgG-HRP (WB, 1:5000)	Santa Cruz Biotechnology Inc, Dallas, TX	Cat# sc-2313; RRID: AB_641181
Goat anti-mouse IgG-HRP (WB, 1:5000)	Santa Cruz Biotechnology	Cat# sc-2005; RRID: AB_631736
Rabbit anti-IP$_3$R1 (WB, 1:1000)	Cell Signaling Technology, Boston, MA	Cat# 3763; RRID: AB_2129958
Rabbit anti-IP$_3$R2 (WB, 1:1000)	Custom-made to peptide (GFLGSNTPHENHHMPPH) by Pocono Rabbit Farm and Laboratory, Inc, Canadensis, PA. (Mataragka and Taylor, 2018)	n/a
Mouse anti-IP$_3$R3 (WB,1:1000)	BD Biosciences, Wokingham, UK	Cat# 610312; RRID: AB_397704
Mouse anti-β-actin (WB, 1:1000)	Cell Signaling Technology	Cat# 3700; RRID: AB_2242334
Rabbit (monoclonal) anti-ORP1 (WB, 1:1000)	Abcam, Cambridge	Cat# ab131165; RRID: AB_11155305
Mouse anti-VAP-A (PLA, 1:100)	Santa Cruz Biotechnology	Cat# sc-293278
Rabbit anti-Rab7 (PLA, 1:100)	Cell Signaling Technology	Cat# 9367; RRID: AB_1904103
Mouse anti-GFP (PLA, 1:500)	ThermoFisher, Paisley, UK	Cat# A-11120; RRID: AB_221568
Rabbit (monoclonal) anti-LAMP1 (PLA, 1:200)	Cell Signaling Technology	Cat# 9091; RRID: AB_2687579
Chemicals, Peptides, and Recombinant Proteins		
ATP disodium salt	Sigma-Aldrich	Cat# A9187
Alexa Fluor^{TM} 488-dextran conjugate (10,000 MW)	ThermoFisher	Cat# D22910
Bafilomycin A_1 (Baf A_1)	Fluorochem, Hadfield, UK	Cat# M01404
Bafilomycin A_1 (Baf A_1)	Alfa Aeser via ThermoFisher	Cat# JS1835
BAPTA	Molekula, Dorset, UK	Cat# 20358510
Bovine serum albumin (BSA)	Europa Bioproducts, Cambridge, UK	Cat# EQBAH64
Caged cell-permeant IP$_3$ (ci-IP$_3$ PM)	SiChem, Bremen, Germany	Cat# cag-iso-2-145-100
Carbachol (carbamoylcholine chloride, CCh)	Sigma-Aldrich, Gillingham, UK	Cat# Y0000113
cOmplete EDTA-free protease inhibitor cocktail	Sigma-Aldrich	Cat# 11873580001
Concanamycin A (CcA)	Insight Biotechnology, Middlesex, UK	Cat# sc-202111A
Cyclopiazonic acid (CPA)	Bio-Techne, Minneapolis, MN	Cat# 1235
Dimethyl sulfoxide (DMSO)	Sigma-Aldrich	Cat# D2650
DMEM/F-12, GlutaMAX medium	ThermoFisher	Cat# 31331028
ECL Prime chemiluminescence detection reagent	GE Healthcare, Little Chalfont, UK	Cat# RPN2232
Fetal bovine serum (FBS)	Sigma-Aldrich	Cat# F7524, batch 094M3341
Fibronectin (human)	Merck Millipore, Watford, UK	Cat# FC010
Fluorescein-conjugated dextran (10,000 MW, Fluoro-Emerald)	ThermoFisher	Cat# D1820
Fluo 8-AM	Stratech Scientific, Suffolk, UK	Cat# 21080-AAT
HEPES	Merck Millipore	Cat# 391338
Histamine dihydrochloride	Sigma-Aldrich	Cat# H7250
Inositol 1,4,5-trisphosphate (IP$_3$)	Enzo, Exeter, UK	Cat# BML-CA430-0001
Ionomycin	Apollo Scientific, Stockport, UK	Cat# 56092-81-0
Iscove's Modifided Dulbecco's Medium (IMDM) with GlutaMAX	ThermoFisher	Cat# 12440-05
LysoTracker Red DND-99	ThermoFisher	Cat# L7528
Mag-fluo 4-AM	Cambridge Bioscience, Cambridge, UK	Cat# M-14206

(*Continued on next page*)

Continued

REAGENT OR RESOURCE	SOURCE	IDENTIFIER
NucBlue Live Ready Probe	ThermoFisher	Cat# R37606
PIPES	Sigma-Aldrich	Cat# P1851
Pluronic F-127	Sigma-Aldrich	Cat# P2443
Poly-L-lysine	Sigma-Aldrich	Cat# P8920
Restriction enzyme: BamHI	Fermentas via ThermoFisher	Cat# FD0054
Restriction enzyme: EcoRI	Fermentas via ThermoFisher	Cat# FD0274
Restriction enzyme: HindIII	Fermentas via ThermoFisher	Cat# FD0504
Saponin	Sigma-Aldrich	Cat# S4521
siPORT NeoFX transfection reagent	ThermoFisher	Cat# AM4511
T4 DNA ligase	ThermoFisher	Cat# M0202S
Thapsigargin	Bio-Techne, Minneapolis, MN	Cat# 1138
TransIT-LT transfection reagent	GeneFlow, Lichfield, UK	Cat# E7-0002
Tris base	ThermoFisher	Cat# BP152-1
Triton X-100	Sigma-Aldrich	Cat# T8787
TrypLE Express	ThermoFisher	*Cat# 12605010*
Tween-20	Sigma-Aldrich	Cat# T5927
U73122	Bio-Techne	Cat# 1268/10
U73343	Bio-Techne	Cat# 4133/10
Critical Commercial Assays		
DC™ protein assay kit II	BioRad, Watford, UK	Cat# 5000112
Duolink *in situ* Red Starter kit mouse/rabbit	Sigma-Aldrich	Cat# DUO92101-1KT
Duolink *in situ* PLA probe: anti-rabbit PLUS, affinity-purified donkey anti-rabbit IgG	Sigma-Aldrich	Cat# DUO92002-100RXN
Duolink *in situ* PLA probe: anti-mouse MINUS, affinity-purified donkey anti-mouse IgG	Sigma-Aldrich	Cat# DUO92004-100RXN
Duolink *in situ* detection reagents Red	Sigma-Aldrich	Cat# DUO92008-100RXN
Duolink *in situ* mounting medium	Sigma-Aldrich	Cat# DUO82040
Duolink *in situ* wash buffer fluorescence	Sigma-Aldrich	Cat# DUO82049
FastLane cell cDNA kit	QIAGEN, Crawley, UK	Cat# 215011
Plasmid maxi kit	QIAGEN	Cat# 12165
QIAquick gel extraction kit	QIAGEN	Cat# 28706
QIAprep spin miniprep kit	QIAGEN	Cat# 27104
QIAprep endoFree plasmid maxi kit	QIAGEN	Cat# 12362
Rotor-Gene SYBR Green PCR kit	QIAGEN	Cat# 204074
Experimental Models: Cell Lines		
EGFP-IP$_3$R1-HeLa cells	(Thillaiappan et al., 2017)	n/a
HAP1 cells without IP$_3$Rs	Horizon Discovery, Cambridge, UK<br>This study	n/a
HEK cells	Dr David Yule, University of Rochester, NY (parental cell line from which HEK cells lacking IP$_3$R subtypes were generated); (Alzayady et al., 2016)	n/a
HEK cells expressing single native IP$_3$R subtypes	Kerafast, Boston, MA (cell lines generated by Dr Yule); (Alzayady et al., 2016)	Cat# EUR031, EUR032, EUR033, EUR034, EUR035 and EUR036
Oligonucleotides		
Silencer™ siRNA against human ATP6V0C	ThermoFisher	Cat# 4390824
Silencer™ Select siRNA against human ORP1L (also known as OSBPL1A)	ThermoFisher	Cat# s41681 and s41682

(*Continued on next page*)

REAGENT OR RESOURCE	SOURCE	IDENTIFIER
Continued		
Silencer negative control No.1 siRNA	ThermoFisher	Cat# AM4611
Primers for sequencing and construction of plasmids, see Table S1.	ThermoFisher This paper	n/a
QuantiTect QPCR primer for human GAPDH	QIAGEN	Cat# QT00079247
QuantiTect QPCR primer for human ATP6V0C	QIAGEN	Cat# QT00220738
Recombinant DNA		
pcDNA3.1(+) plasmid	ThermoFisher	Cat# V790-20
G-GECO1.2 (CyGG)	Addgene (Zhao et al., 2011)	Cat# 32446
LAMP1-mCherry	(López Sanjurjo et al., 2013)	n/a
LAMP1-G-GECO1.2 (Ly-GG) in pcDNA3.1(+)	This study	n/a
mTurquoise-LAMP1 (we note that despite the nomenclature, the LAMP1 is tagged at its C terminus with mTurquoise in this construct)	Addgene, deposited by Michael Davidson, Florida State University	Cat# 55568
mCherry-ER	Addgene, deposited by Michael Davidson, Florida State University	Cat# 55041
TPC2-mRFP	(Brailoiu et al., 2009)	n/a
TPC2-GFP	(Brailoiu et al., 2009)	n/a
mRFP-ORP1L and variants, see Figure S6:	Dr J Neefjes (University of Leiden Medical Center, the Netherlands	n/a
ΔORD	(Rocha et al., 2009)	n/a
mRFP-ΔORDPHDPHD		n/a
mRFP-ΔORD		n/a
Software and Algorithms		
BioEdit, version 7.0.5	Ibis Therapeutics, North Carolina State University, NC	http://www.mbio.ncsu.edu/BioEdit
CellProfiler, version 2.1	n/a	http://cellprofiler.org
Clustal Omega	n/a	https://www.ebi.ac.uk/Tools/msa/clustalo
Fiji/ImageJ	(Schindelin et al., 2012)	http://fiji.sc/
GeneTools, version 4	Syngene, Cambridge, UK	https://www.syngene.com/
MetaMorph Microscopy Automation and Image Analysis	Molecular Devices, San Jose, CA	https://www.moleculardevices.com/
Prism 5, version 5	GraphPad, La Jolla	https://www.graphpad.com/
SoftMax Pro, version 7	Molecular Devices, San Jose, CA	https://www.moleculardevices.com/

CONTACT FOR REAGENT AND RESOURCE SHARING

Further information and requests for resources and reagents should be directed to and will be fulfilled by the Lead Contact, Colin W. Taylor (cwt1000@cam.ac.uk).

EXPERIMENTAL MODEL AND SUBJECT DETAILS

Cell Culture and Transfection

The methods used to establish EGFP-IP_3R1-HeLa cells, in which all endogenous IP_3R1 are N-terminally tagged with monomeric EGFP have been fully described (Thillaiappan et al., 2017). In brief, we used gene-editing with transcription activator-like effector nucleases (TALENs) to modify both copies of the IP_3R1 gene. Sequencing alongside functional and optical microscopy analyses, confirmed both the selectivity of the editing and that the edited IP_3Rs form functional Ca^{2+} release channels (Thillaiappan et al., 2017). HEK cells, in which CRISPR/Cas9 was used to delete one or more IP_3R subtypes, were generated by Dr David Yule's laboratory (University of Rochester, NY) (Alzayady et al., 2016) and supplied by Kerafast. HAP1 cells, genetically engineered using CRISPR/Cas9 to disrupt genes for all three IP_3R subtypes, were developed in collaboration with Horizon Discovery (Cambridge, UK). Short tandem repeat profiling was used to authenticate the HeLa cells used (Eurofins, Germany) and the HEK cells lacking

all three IP_3R subtypes (Public Health England). We have not confirmed the authenticity of the HAP1 cell lines. Regular screening throughout the study established that all cell lines were free of mycoplasma.

HeLa and HEK293 cell lines were cultured in Dulbecco's Modified Eagles Medium (DMEM)/F-12 with GlutaMAX supplemented with fetal bovine serum (FBS, 10%). HAP1 cells were cultured in Iscove's Modified Dulbecco's Medium (IMDM) GlutaMAX with 10% FBS. All cells were maintained at 37°C in humidified air with 5% CO_2, and passaged every 3-4 days using TrypLE Express.

For imaging, cells were grown on 35-mm glass-bottomed dishes (#P35G-1.0-14-C, MatTek Corporation, Ashland, MA, USA; or D35-14-1-N, IBL Baustoff+Labor, Austria) coated with human fibronectin (10 μg/ml). Cells were transfected with plasmids encoding Ca^{2+} indicators (Ly-GG or Cy-GG) or tagged proteins according to the manufacturer's instructions using TransIT-LT1 reagent (1 μg DNA/2.5 μl reagent). For siRNA transfections, cells were plated in clear-bottomed 96-well plates (Greiner Bio-One, Stonehouse, UK) coated with poly-L-lysine (0.01% w/v). After 24 hr, cells were transfected with Silencer™ siRNA (40 nM) directed against ATP6V0C or a non-silencing control siRNA using siPORT NeoFX transfection reagent (220 ng siRNA/μl reagent). Experiments were performed 48-72 hr after transfection. The same methods were used for transfection with siRNA directed against human ORP1L (Alpy et al., 2013) (Figure S6).

METHOD DETAILS

Plasmids

The genetically-encoded, low-affinity Ca^{2+} sensor G-GECO1.2 (equilibrium dissociation constant for Ca^{2+}, K_D^{Ca} = 1.2 μM) (Zhao et al., 2011) was used to record $[Ca^{2+}]_c$. The initial templates for cloning of a low-affinity Ca^{2+} sensor targeted to the cytosolic surface of the lysosomal membrane (Ly-GG) were LAMP1-mCherry and cytosolic G-GECO1.2. A HindIII recognition site was inserted at the 5′ end of LAMP1-mCherry using primer LAMP1F (the sequences of all primers used and their codes are provided in Table S1). LAMP1-mCherry has a pre-existing BamHI site. Primers LAMP1F and LAMP1R were used to amplify the LAMP1 sequence from LAMP1-mCherry using PCR. The LAMP1 PCR product was digested with HindIII and BamHI. A BamHI site was introduced in-frame with the 5′ end of G-GECO1.2 by PCR using primer G-GECO1.2F, and an EcoRI site was introduced at the 3′ end of G-GECO1.2 using primer G-GECO1.2R. The product was then digested with BamHI and EcoRI. The LAMP1-G-GECO1.2 construct was assembled in the pcDNA3.1(+) expression vector. pcDNA3(+) was digested with HindIII and EcoRI overnight to create sticky ends suitable for ligation with the LAMP1 and G-GECO1.2 fragments. The digested LAMP1, G-GECO1.2 and pcDNA3.1(+) were ligated using T4 DNA ligase according to the manufacturer's protocol. The complete coding sequence of LAMP1-G-GECO1.2 was verified using the following primers: LAMP1SeqF1, LAMP1SeqF2,

T7 promoterF, G-GECO1.2SeqM and GGECO1.2SeqE (Table S1). Sequencing data were analyzed using BioEdit software, and alignments were carried out using Clustal Omega. The cytosolic and lysosome-targeted G-GECO1.2 s are described as Cy-GG and Ly-GG in the text. Plasmids encoding TPC2-mRFP and TPC2-GFP (Brailoiu et al., 2009), LAMP1-GFP (López Sanjurjo et al., 2013), mTurquoise-LAMP1, mCherry-ER and LAMP1-mCherry (López Sanjurjo et al., 2013) have been described. Plasmids encoding ORP1L and its variants (Rocha et al., 2009) (Figure S6) were provided by Dr J Neefjes (University of Leiden Medical Center, the Netherlands).

Measurements of $[Ca^{2+}]_c$ in Cell Populations

Confluent monolayers of cells grown in a 96-well plate (Greiner Bio-One, Storehouse, UK) were loaded with fluo 8 by incubation for 1 hr at 20°C in HEPES-buffered saline (HBS, 100 μl) containing fluo 8-AM (2 μM) and 0.02% Pluronic F-127. Cells were then washed and incubated in HBS for 1 h at 20°C to allow de-esterification of fluo 8-AM. HBS had the following composition: 135 mM NaCl, 5.9 mM KCl, 1.2 mM $MgCl_2$, 1.5 mM $CaCl_2$, 11.5 mM D-glucose, 11.6 mM HEPES, pH 7.3. $CaCl_2$ was omitted from nominally Ca^{2+}-free HBS. In some experiments, BAPTA (final concentration 2.5 mM) was added to HBS immediately before stimulation to reduce the free $[Ca^{2+}]$ of the HBS to < 20 nM. Fluorescence was recorded using a FlexStation III fluorescence plate-reader (Molecular Devices, Sunnyvale, CA, USA) (López Sanjurjo et al., 2013). Fluorescence was recorded at 1.44-s intervals, with excitation at 485 nm and emission at 525 nm. Data were collected and analyzed using SoftMax Pro software. Maximal (F_{max}) and minimal (F_{min}) fluorescence values were determined from parallel wells after addition of Triton X-100 (0.1%) to lyse cells in the presence of either 10 mM $CaCl_2$ (F_{max}) or 10 mM BAPTA (F_{min}). Fluorescence values (F) were calibrated to $[Ca^{2+}]_c$ using a K_D = 389 nM from:

$$[Ca^{2+}]_c = K_D \times \frac{(F - F_{min})}{(F_{max} - F)}$$

IP_3-evoked Ca^{2+} release from saponin-permeabilized HAP1 cells was measured in cytosol-like medium (CLM) using a low-affinity Ca^{2+} indicator (Mag-fluo 4) trapped within the ER, as previously described for other cell types (Tovey et al., 2006). Briefly, cells were loaded with the indicator by incubation with 20 μM Mag-fluo 4-AM in HBS containing BSA (1 mg/ml) and pluronic acid (0.02%, v/v). After 1 hr at 20°C, cells were resuspended in Ca^{2+}-free CLM, which had the following composition: 140 mM KCl, 20 mM NaCl, 1 mM EGTA, 2 mM $MgCl_2$ and 20 mM PIPES, pH 7.0. The plasma membrane was then permeabilized by incubation with saponin (10 μg/ml, 2-3 min, 37°C). Cells were recovered (600 x*g*, 2 min), re-suspended (~10^7 cells/ml) in Mg^{2+}-free CLM, distributed (45 μl/well) into black half-area 96-well plates and centrifuged (300 x*g*, 2 min). Mag-fluo 4 fluorescence (excitation at 485 nm, emission at 525 nm) was recorded at 1.44-s intervals at 20°C using a FlexStation III fluorescence plate-reader. Addition of MgATP

(1.5 mM) allowed Ca^{2+} uptake into the intracellular stores. When steady-state Ca^{2+} loading was achieved (~2 min), IP_3 was added with cyclopiazonic acid (CPA, 10 μM) to inhibit further Ca^{2+} uptake. IP_3-evoked Ca^{2+} release is reported as the fractional decrease in the ATP-dependent Mag-fluo 4 fluorescence.

Fluorescence Microscopy

Fluorescence microscopy used an inverted Olympus IX83 microscope equipped with a 100x objective (numerical aperture, NA, 1.49), a multi-line laser bank (405, 425, 488, 561 and 647 nm) and an $iLas^2$ targeted laser illumination system (Cairn, Faversham, UK). Excitation light was transmitted through either a quad dichroic beam-splitter (TRF89902-QUAD) or a dichroic mirror (for 425 nm; ZT442rdc-UF2, Chroma, Germany). Emitted light was passed through appropriate filters (Cairn Optospin; peak/bandwidth: 450/50, 480/40, 525/50, 630/75 and 700/75 nm) and detected with either an iXon Ultra 897 electron multiplied charge-coupled device (EMCCD) camera (512 × 512 pixels, Andor, Belfast, Northern Ireland) or (for Figures 4B–4E and S2–S4) a Prime 95B Scientific Complementary Metal Oxide Semiconductor (sCMOS) camera (1200 × 1200 pixels, Photometrics, Tucson, AZ, USA). For all multi-color imaging, we confirmed that there was no bleedthrough between channels. For TIRFM, the penetration depth was 90-140 nm. The $iLas^2$ illumination system was used for TIRFM and wide-field imaging. Bright-field images were acquired using a Cairn MonoLED illuminator. All fluorescence images were corrected for background by subtraction of fluorescence collected from a region outside the cell. Image capture and processing used MetaMorph Microscopy Automation and Image Analysis software.

For colocalization analyses, we used either Pearson's correlation coefficient (R_{coloc}) for comparisons of fluorophores in every pixel, or Manders' split coefficient to identify the fraction of PLA spots that colocalized with EGFP-IP_3R1 (Figure 5D). We confirmed, using the Costes randomization method with 100 iterations and ignoring pixels in which there was no fluorescence (Costes et al., 2004), that any colocalization was more than expected from randomly distributed fluorophores (ImageJ Colocalization Analysis/Colocalization Test). R_{coloc} was calculated from all pixels within the region of interest (ROI) that exceeded a threshold value (ImageJ Colocalization Analysis/Colocalization Threshold):

$$R_{coloc} = \frac{\sum(Ri - Rm)(Gi - Gm)}{\sqrt{\sum(Ri - Rm)^2 \sum(Gi - Gm)^2}}$$

where, Gi and Ri are the intensities of individual green and red pixels respectively, and Gm and Rm are the mean intensities of green and red pixels.

To measure center-center distances between each lysosome (mTurquoise-LAMP1) and the nearest EGFP-IP_3R1 punctum (Figures 7G and 7H), images were Gaussian-filtered to remove noise, and then analyzed using the ImageJ Distance Analysis plug-in (DiAna) (Gilles et al., 2017).

Measurement of Near-Lysosome Ca^{2+} Signals

HeLa cells transfected with Ly-GG were washed three times in HBS, and Ly-GG fluorescence (excitation at 488 nm, emission at 525 nm) was imaged using wide-field fluorescence microscopy (1 frame/s) at 20°C. After background correction, the Ly-GG fluorescence associated with single lysosomes was measured using single-particle tracking with the MetaMorph Track Objects plugin (Meijering et al., 2012). A template-match algorithm was used to connect tracks between successive frames. Tracks that terminated before completion of the recording (240 s for cells stimulated with histamine; 1330 s for analyses of SOCE) were excluded from the analysis. In parallel analyses of HeLa cells expressing Cy-GG, ROIs similar in dimensions to tracked lysosomes (radius ~1.6 μm) were selected for analysis.

Photolysis of Caged-IP_3

HeLa cells grown on fibronectin-coated glass-bottomed dishes were first transfected with Ly-GG or Cy-GG (1 μg/μl, 24 hr), then loaded with ci-IP_3/PM (1 μM, 50 min) (Dakin and Li, 2007). After washing and incubation in HBS for a further 45 min, cells were imaged (20°C) using an inverted Olympus IX83 microscope equipped with a 100x objective. Ly-GG fluorescence was recorded in widefield (488 nm excitation, 525/50 nm emission). Cells were imaged for 50 s before photolysis of ci-IP_3 using a SPECTRA X-light engine (Lumencor, 395/20 excitation, exposure time 50 ms/frame for 10 frames). Images were acquired at 50-ms intervals with an iXon Ultra 897 EMCCD camera, corrected for background fluorescence, and analyzed using MetaMorph. Ly-GG was tracked to determine Ca^{2+} signals around single lysosomes. Photolysis of ci-IP_3 releases an active, but more metabolically stable, analog of IP_3 (i-IP_3, in which the 2- and 3-hydroxyls are linked by an isopropylidene group) (Dakin and Li, 2007).

Measurement of Lysosomal pH

The pH within lysosomes was measured from defined ROI within single cells using a dextran-conjugated ratiometric pH indicator, fluorescein-dextran, loaded into lysosomes by endocytosis. Cells were incubated with fluorescein-dextran (10-kDa, 0.2 mg/ml) for 16 hr, followed by a 4-hr chase in DMEM F12 at 37°C. The cells were then washed 3 times with HBS, and imaged immediately with alternating excitation/emission (F_{425}: λ_{ex} = 425 nm, λ_{em} = 480 nm. F_{488}: λ_{ex} = 488 nm, λ_{em} = 525 nm). Images were collected for 100 ms, with 5 min between each round of data acquisition. After background subtraction, ROIs were drawn around lysosome

clusters and fluorescence ratios (R, which increases with increased pH) were calculated from F_{488}/F_{425} at each time. Results are presented as R/R_0, where R_0 is the fluorescence ratio recorded before stimulation.

For experiments with LysoTracker Red, cells were loaded with 50 nM LysoTracker Red DND-99 for 1 hr, washed 3 times with HBS, and imaged immediately with excitation and emission at 561 nm and 630 nm, respectively.

Measurement of Lysosome Size

Lysosome size was measured in HEK cells using either LAMP1-mCherry (Figures 7E and 7F) or endocytosed Alexa Fluor 488-dextran (10,000, MW) (Figure S7) to identify lysosomes. After application of a threshold (ImageJ Threshold), particles were accepted for analysis if they had a circularity value ($4.\pi$.area/circumference^2) of 0.6-1.0. The circularity criterion ensured that only roughly circular particles were selected for analysis (Grossi et al., 2016). Visual inspection of images before and after application of the selection criteria confirmed that most lysosomes were included in the final analysis. We use the Feret diameter to report lysosome size, which is the maximum distance between two points on the perimeter of the particle (ImageJ Analyze Particles) (Ferraro et al., 2014).

Quantitative PCR

QPCR was carried out as previously described (Tovey et al., 2008). cDNA was synthesized in a final volume of 20 μl from a lysate prepared from confluent cells in 1 well of a 96-well plate, using a FastLane cell cDNA kit. For QPCR, each reaction included primers for ATP6V0C and, for calibration, primers for a housekeeping gene (glyceraldehyde phosphate dehydrogenase, GAPDH). Each reaction (20 μl) included Rotor-Gene SYBR Green PCR master mix (10 μl), cDNA (5 μl), Quantitect primer assay (2 μl) and RNAase-free water (3 μl). In two negative controls, the primers were omitted during QPCR, or the reverse transcriptase was omitted during cDNA synthesis. For QPCR (Rotor-Gene 6000, Corbett Life Sciences), an initial denaturation at 95°C for 5 min was followed by 40 cycles of amplification (93°C for 5 s, 60°C for 10 s) and then a melting curve (72°C to 95°C). Expression of mRNA relative to that for GAPDH was calculated from:

$$\text{Expression} = \frac{E^{-C_T^{ATP6V0C}}}{E^{-C_T^{GAPDH}}}$$

E is the amplification efficiency, calculated as 10^m, where m is the average fluorescence increase for the four cycles after the cycle threshold (C_T) for the indicated PCR product. Results are reported as mean ± SD for cDNA samples independently isolated from 3 different experiments.

Proximity Ligation Assays

A Duolink proximity ligation assay (PLA) was used to quantify interactions between proteins less than ~40 nm apart, according to the manufacturer's instructions. The method uses antibodies from two species (mouse and rabbit) to recognize two candidate proteins *in situ*. The antibodies are then recognized by secondary antibodies conjugated to complementary oligonucleotides, which are amplified to incorporate a fluorescent nucleotide (Texas Red) only if the pair of secondary antibodies are less than ~40 nm apart (Fredriksson et al., 2002; Koos et al., 2014) (Figure 5A).

Cells gown on fibronectin-coated 35-mm glass-bottom dishes were fixed at 20°C (4% paraformaldehyde, 30 min), washed with PBS, permeabilized (0.25% Triton X-100, 5 min), and incubated with primary antibodies (16 hr, 4°C). For ER-lysosome interactions, the primary antibodies were against VAP-A (ER) and either LAMP1 or Rab7 (both lysosomes), and for EGFP-IP$_3$R1-lysosome interactions they were against GFP and either LAMP1 or Rab7. Incubations with Duolink PLA probe (anti-rabbit PLUS and anti-mouse MINUS), ligase and polymerase, and the washes between each step, were exactly as recommended by the manufacturer. Cells were then mounted in Duolink II mounting medium containing DAPI to label the nucleus. PLA products were visualized using an Olympus microscope with x60 or x100 objective, and spots were quantified using CellProfiler software. The specificity of the PLA reactions was confirmed by omission of either primary antibody (VAP-A or Rab7) and, for EGFP-IP$_3$R1-lysosome measurements by using cells without EGFP-IP$_3$R1 (Figure 5).

Western Blots

Cells isolated by centrifugation (600 x*g*, 2 min) were lysed in cold medium containing protease inhibitors (cOmplete, EDTA-free Protease Inhibitor Cocktail) and the supernatant (900 x*g*, 15 min) was used for western blotting. Proteins were separated (4%–8% RunBlue SDS gel, Expedeon, San Diego, CA), transferred to a polyvinyl difluoride (PVDF) membrane using an iBLOT gel-transfer system (ThermoFisher), blocked in Tris-buffered saline (50 mM Tris-HCl, 150 mM NaCl, pH 7.5) containing 0.2% Tween-20 and 5% BSA for 1 hr, washed in the same medium, and incubated with primary antibody (16 hr, 4°C) in the blocking buffer. After washing (3 × 5 min), the membrane was incubated with secondary antibody (1 hr, 20°C), washed (3 × 5 min). Bands were visualized using ECL Prime western blotting detection reagent and a Syngene Pxi chemiluminescence detection system with GeneTools software.

QUANTIFICATION AND STATISTICAL ANALYSIS

We did not use power analyses to determine sample sizes. In all assays using multi-well plates, the positions of treatments were varied to avoid place-dependent systematic errors.

All statistical analyses used Prism, version 5. For analyses of concentration-effect relationships, non-linear curve-fitting to a Hill equation was used to provide values for pEC_{50} (-log of the half-maximally effective concentration) and maximal response for each individual experiment. The individually determined pEC_{50} values were then pooled for statistical analysis.

All results are presented as mean ± SD or SEM, as appropriate. Student's t test (for 2 variables), and one-way or two-way ANOVA with Tukey's multiple comparison test or Bonferroni post hoc test (more than 2 variables) were used for statistical analyses. The Kolmogorov-Smirnov normality test was used to determine whether frequency distributions deviated from normality ($p < 0.05$) (Figures 2F and 2G). Sample sizes (*n*) refer to independent experiments. $p < 0.05$ was considered significant. The tests used are reported in the figure legends.

Cell Reports, Volume *25*

Supplemental Information

IP_3 Receptors Preferentially Associate with ER-Lysosome Contact Sites and Selectively Deliver Ca^{2+} to Lysosomes

Peace Atakpa, Nagendra Babu Thillaiappan, Stefania Mataragka, David L. Prole, and Colin W. Taylor

Table S1 Primers Used for Constructing and Sequencing Plasmids. Related to STAR methods.

Primer name	Sequence
GGECO1.2F	CATGGATCCATGGTCGACTCATCACGTCGTAAG
GGECO1.2R	GTAGAATTCCTACTTCGCTGTCATCATTTG TACAAACTCTTC
LAMP1F	TACAAGCTTGCTTCGAATTCTCGCCACCAT
LAMP1R	GGTGGATCCTCCTGAACCTCCGATGGTCTG ATAGCCCGCG
LAMP1SeqF1	ACGTTCAGCACCTCCAACTA
LAMP1SeqF2	ATCGGCAGGAAGAGGAGTCA
T7 promoter (F)	TAATACGACTCACTATAGGG
GGECO1.2SeqM	CAAACCCCAGTGTGTCCAAG
GGECO1.2SeqE	GCCTACCACTACCAGCAGAA

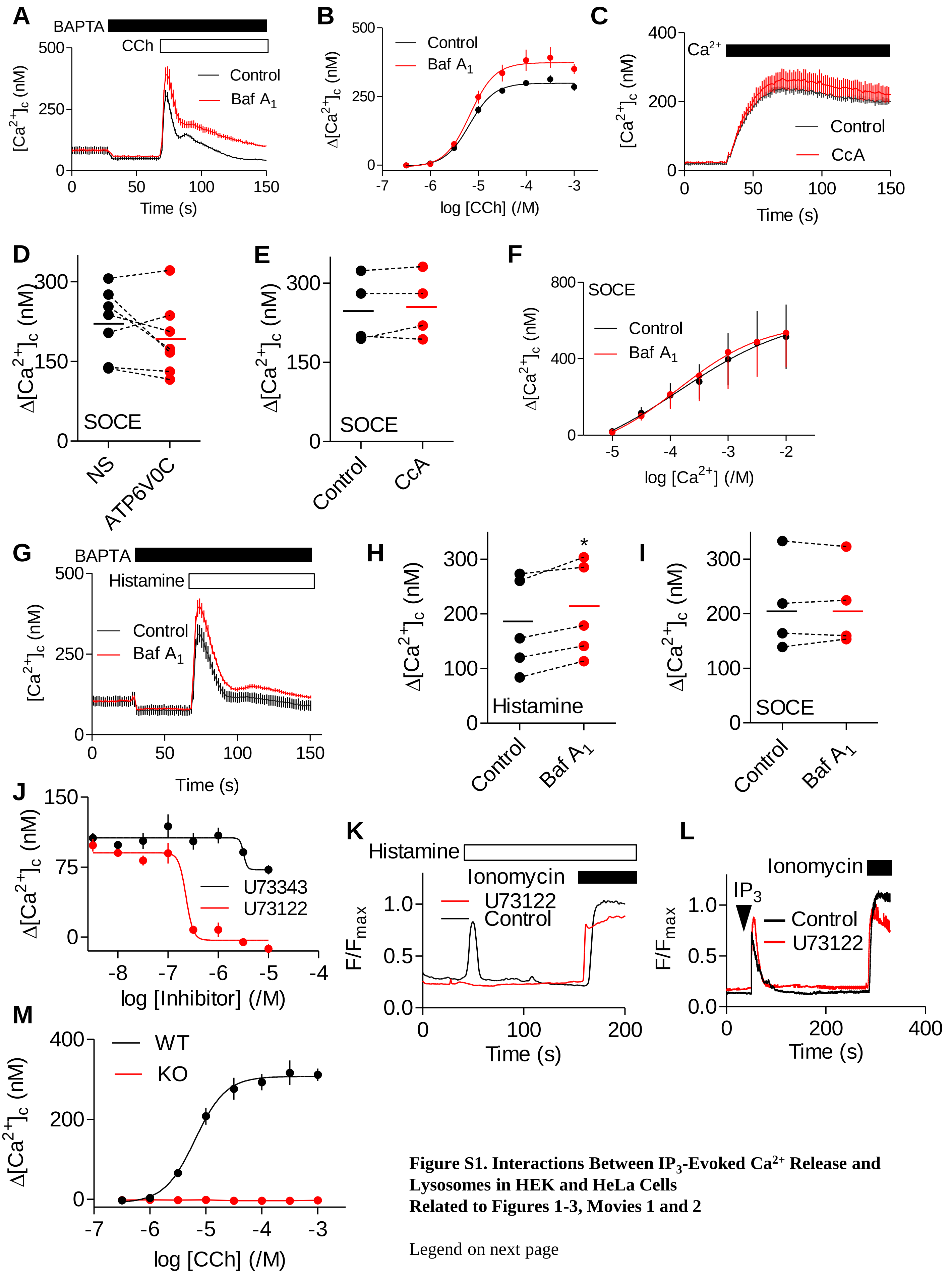

Figure S1. Interactions Between IP$_3$-Evoked Ca^{2+} Release and Lysosomes in HEK and HeLa Cells
Related to Figures 1-3, Movies 1 and 2

Legend on next page

Figure S1. Interactions Between IP_3-Evoked Ca^{2+} Release and Lysosomes in HEK and HeLa Cells

Figure on preceding page

(A) Fluo 8-loaded HEK cells were treated with bafilomycin A_1 (Baf A_1, 1 µM, 1 hr) in HBS before addition of BAPTA (2.5 mM) to chelate extracellular Ca^{2+} and then CCh (1 mM) to stimulate IP_3 formation. Typical results show means ± SD from 3 wells in a single experiment.
(B) Summary results (mean ± SEM, $n = 5$) show effects of CCh on Ca^{2+} release with and without Baf A_1.
(C) HEK cells were treated with thapsigargin (1 µM, 15 min) in Ca^{2+}-free HBS to activate SOCE, before restoration of extracellular Ca^{2+} (10 mM) alone or after treatment with CcA (1 µM, 1 hr). Typical results show mean ± SD from 3 wells in a single experiment.
(D,E) Summary results show $\Delta[Ca^{2+}]_c$ after restoration of extracellular Ca^{2+} to cells treated with siRNA or CcA, as paired comparisons (each with 3 replicates) and the mean value ($n = 7$ (D) or 3 (E), line). $P = 0.18$ (D) and 0.42 (E), paired Student's *t*-test.
(F) HEK cells pre-incubated with Baf A_1 (1 µM, 1 hr) were treated with thapsigargin (1 µM, 15 min) in Ca^{2+}-free HBS before restoration of the indicated concentrations of extracellular Ca^{2+}. Summary results show mean ± SEM, $n = 3$, each with 3 determinations. For clarity, only a single error bar is shown for each mean.
(G) Fluo 8-loaded HeLa cells were treated with Baf A_1 (1 µM, 1 hr) in HBS before addition of BAPTA (2.5 mM) and then histamine (100 µM) to stimulate IP_3 formation. Typical results show mean ± SD from 3 wells in a single experiment.
(H) Summary results show $\Delta[Ca^{2+}]_c$ as paired comparisons (each with 3 replicates) and the mean value ($n = 5$, line). $^{*}P < 0.05$, paired Student's *t*-test.
(I) HeLa cells pre-incubated with Baf A_1 (1 µM, 1 hr) were treated with thapsigargin (1 µM, 15 min) in Ca^{2+}-free HBS before restoration of extracellular Ca^{2+} (10 mM). Paired comparisons ($n = 4$, with 3 determinations in each) are presented in the same format as panel H ($P = 0.81$, paired Student's *t*-test).
(J) Effects of the indicated concentrations of U73122 or U73343 (20 min) on the peak increase in $[Ca^{2+}]_c$ evoked by histamine (100 µM). Results are mean ± SEM from 3 experiments, each with 3 determinations.
(K,L) Typical traces from HeLa cells expressing Ly-GG show the responses to histamine (100 µM) (K) or photolysis of ci-IP_3 (L) in Ca^{2+}-free HBS with or without U73122 (10 µM, 20 min), and then ionomycin (10 µM) with 2 mM $CaCl_2$. Results show responses of a single tracked lysosome. Summary results in **Figure 1L**.
(M) Effects of CCh on Ca^{2+} release in wild-type (WT) HEK cells and HEK cells lacking IP_3Rs (KO). Mean ± SEM, $n = 3$.

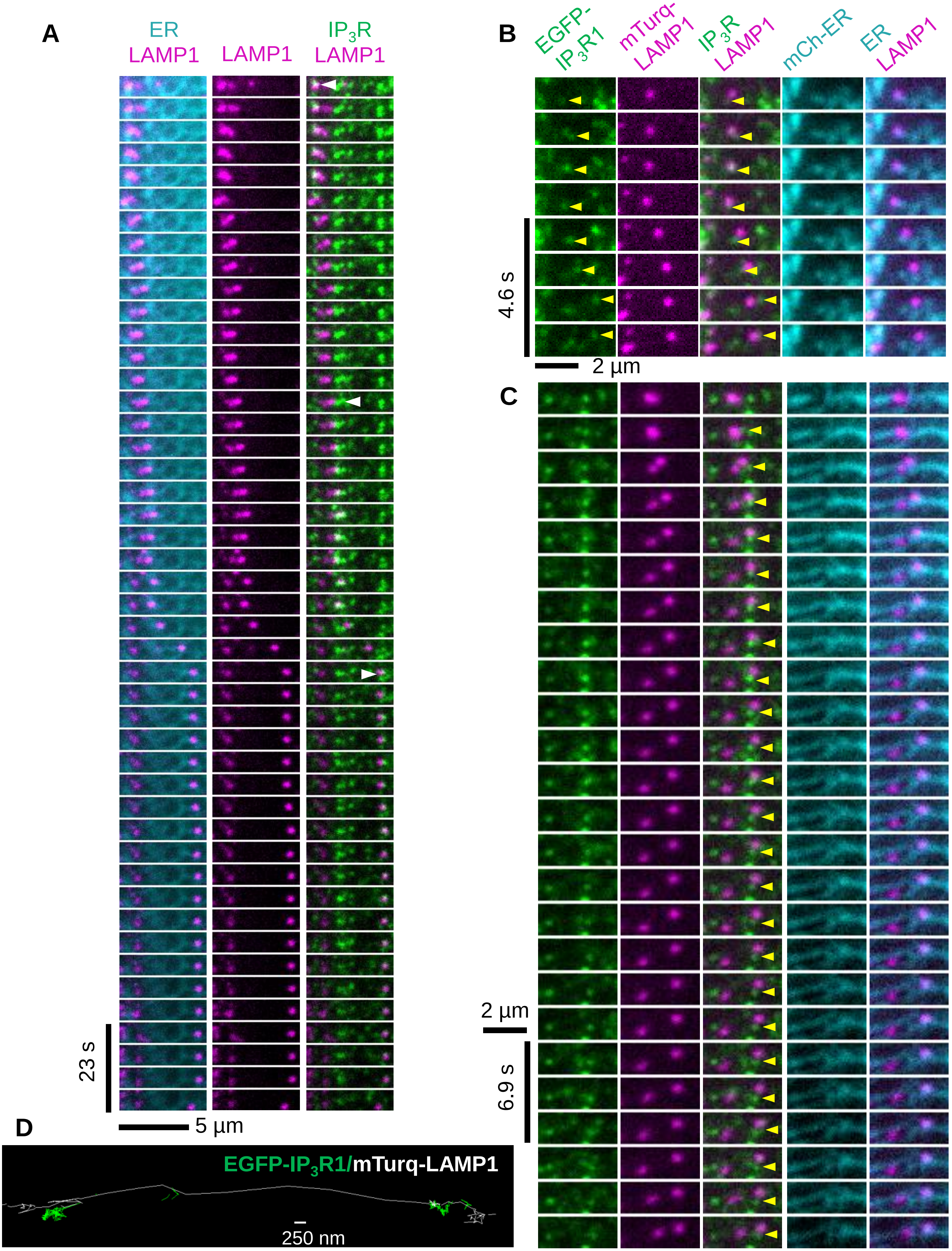

Figure S2. Lysosomes Preferentially Linger at IP$_3$R Puncta: Example 1. Related to Figures 4 and 5, Movies 5 and 6
Legend on next page

Figure S2. Lysosomes Preferentially Linger at IP_3R Puncta: Example 1

Figure on preceding page

TIRFM images of EGFP-IP_3R1-HeLa cells expressing markers of lysosomes (mTurquoise-LAMP1, pseudo-colored in magenta) and the ER lumen (mCherry-ER, pseudo-colored in cyan) were used to construct the kymograms.
(A) A lysosome moves between three different immobile IP_3R puncta (arrows), parking at each for tens of seconds, but moving rapidly along the ER between puncta (see **Movie 5**). White regions indicate colocalization of LAMP1 (magenta) and EGFP-IP_3R1 (green).
(B) A lysosome collides with an IP_3R punctum, and the two then move together. Arrows show positions of the moving IP_3R punctum.
(C) Two lysosomes separate, and one then associates with a slowly moving IP_3R punctum (arrow) with which it then moves for at least 50 s (from **Movie 6**).
(D) Single-particle trajectories (190 s) of two immobile EGFP-IP_3R1 puncta (green) and a lysosome (white) show that the lysosome pauses near the IP_3R puncta, but moves rapidly between them.

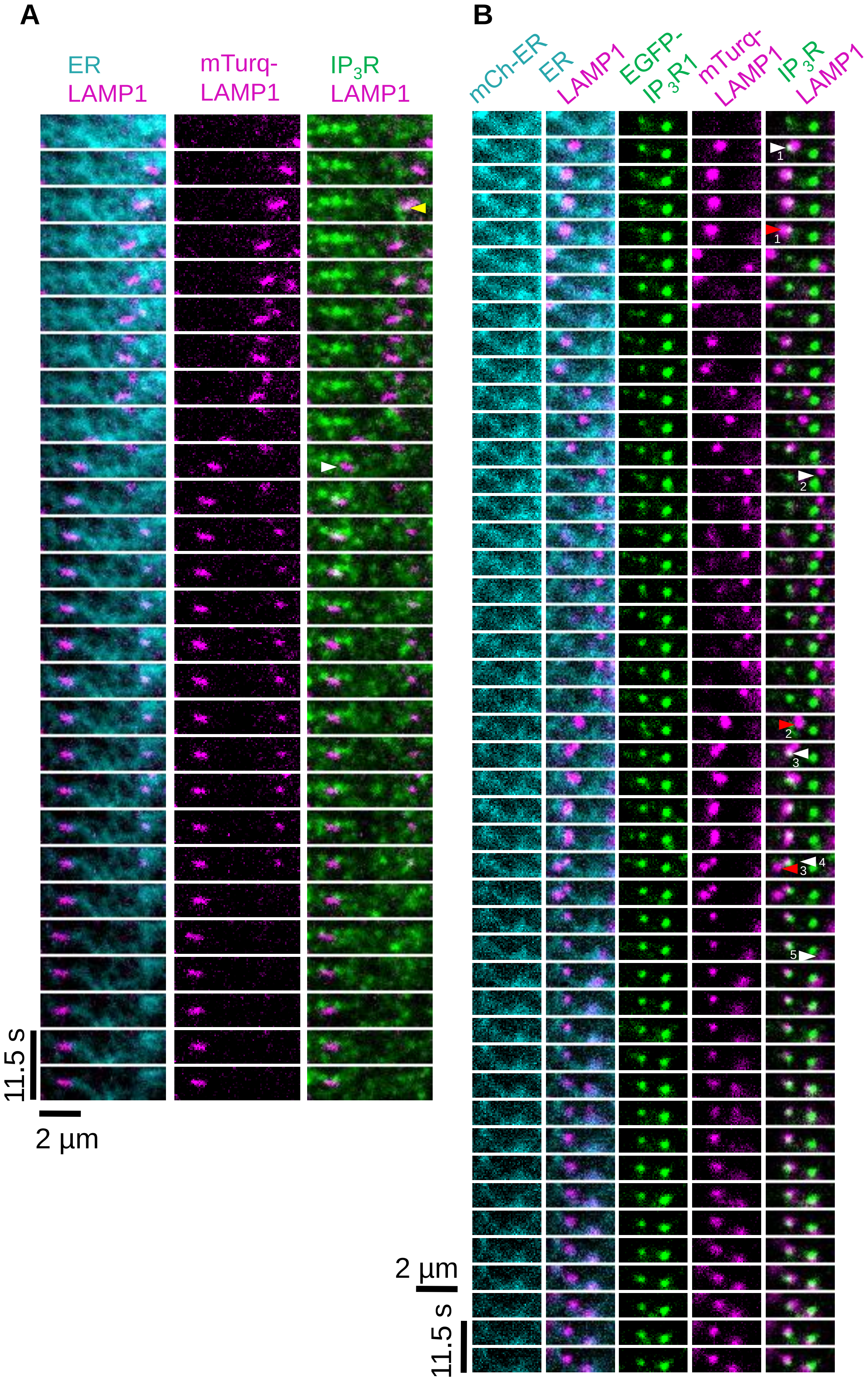

Figure S3. Lysosomes Preferentially Linger at IP_3R Puncta: Example 2. Related to Figures 4 and 5, Movies 5 and 6
Legend on next page

Figure S3. Lysosomes Preferentially Linger at IP_3R Puncta: Example 2

Figure on preceding page

TIRFM images of EGFP-IP_3R1-HeLa cells expressing markers of lysosomes (mTurquoise-LAMP1, pseudo-colored in magenta) and the ER lumen (mCherry-ER, pseudo-colored in cyan) were used to construct kymograms.
(A) Two lysosomes associated with an IP_3R punctum (yellow arrow) separate, with one leaving to join another IP_3R punctum (white arrow).
(B) Each of the two immobile IP_3R puncta shown receives several long-lasting visits by lysosomes (1-5, from **Movie 7**). Each arrival (white arrow) and departure (red arrow) is shown; encounters 4 and 5 persist beyond the recording.

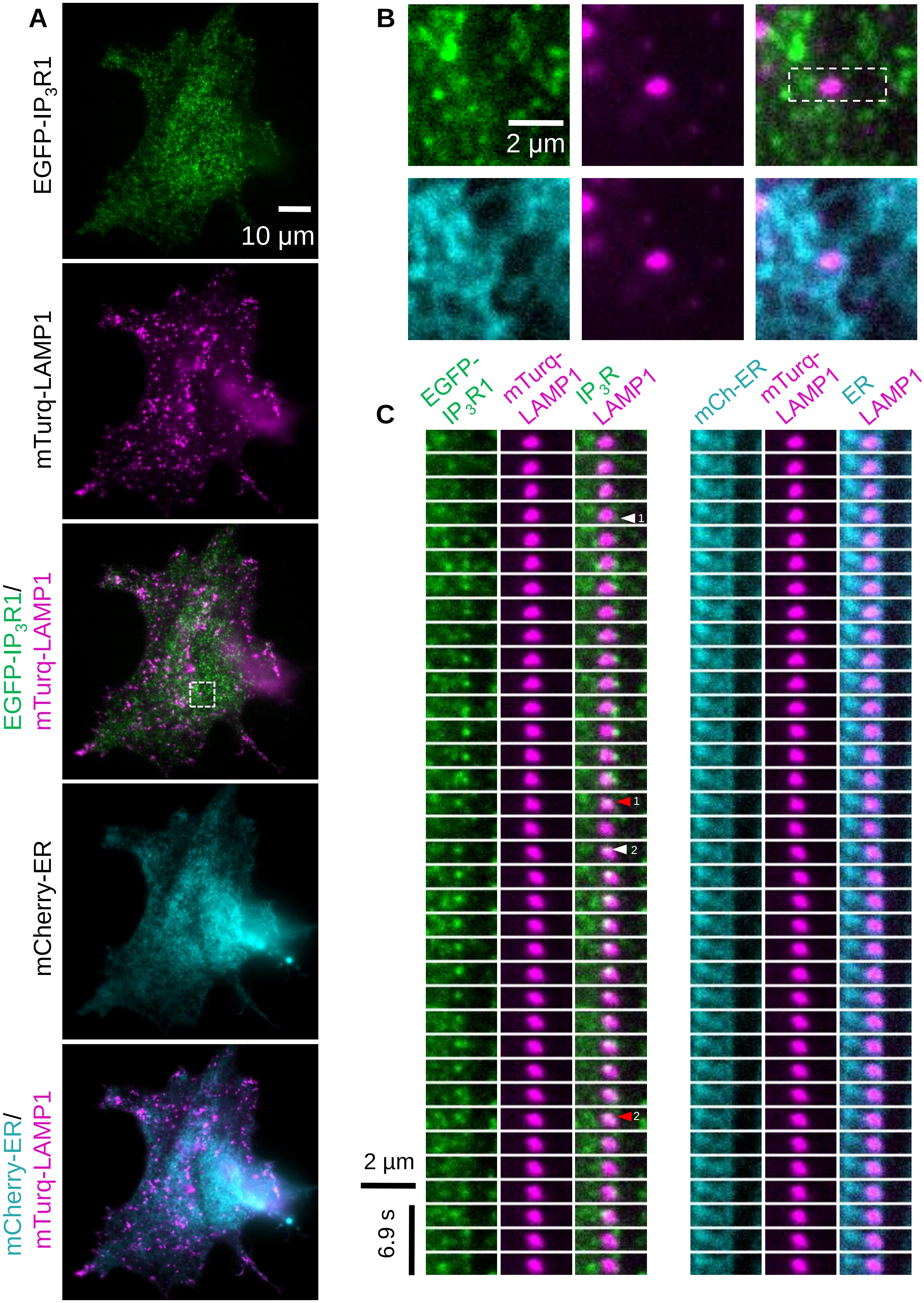

Figure S4. Several Mobile IP$_3$R Puncta Sequentially Associate With a Single Lysosome. Related to Figure 4, Movie 8
Legend on next page

Figure S4. Several Mobile IP_3R Puncta Sequentially Associate With a Single Lysosome

Figure on preceding page

(A) TIRFM images of an EGFP-IP_3R1-HeLa cell expressing markers of lysosomes (mTurquoise-LAMP1, pseudo-colored in magenta) and the ER lumen (mCherry-ER, pseudo-colored in cyan) (from **Movie 8**).
(B) Enlargements of the boxed region in panel A.
(C) Kymograms (2.3-s intervals) from boxed area in panel B show several mobile IP_3R puncta sequentially parking at a single lysosome. Arrows indicate the arrival (white) and departure (red) of two mobile IP_3R puncta.

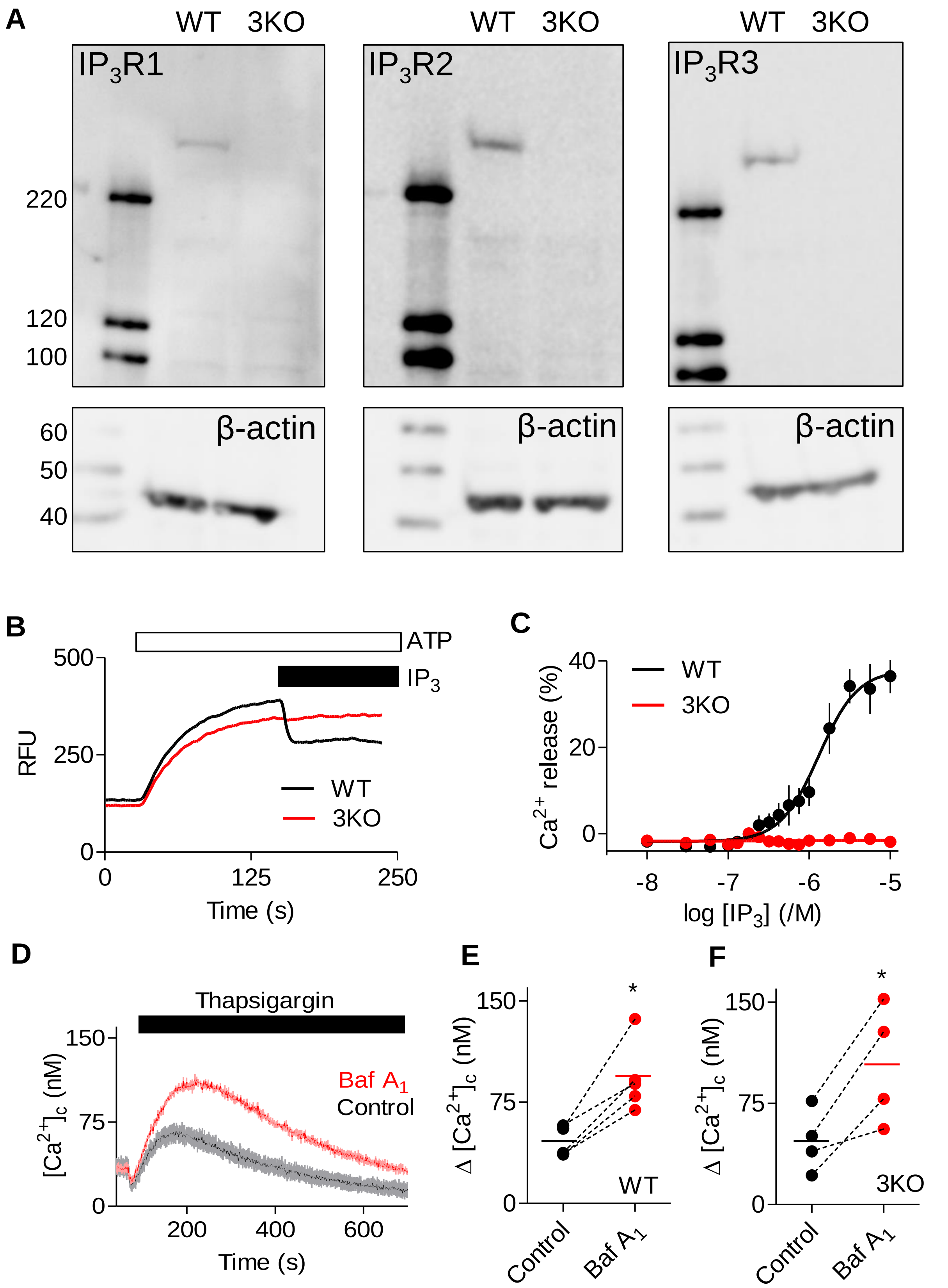

Figure S5. Bafilomycin A_1 Exaggerates Thapsigargin-Evoked Increases in $[Ca^{2+}]_c$ in HAP1 Cells Lacking IP_3Rs
Related to Figure 6, Movies 9 and 10
Legend on next page

Figure S5. Bafilomycin A_1 Exaggerates Thapsigargin-Evoked Increases in $[Ca^{2+}]_c$ in HAP1 Cells Lacking IP_3Rs

Figure on preceding page

CRISPR/Cas9 was used to disrupt the endogenous genes encoding all three IP_3R subtypes in HAP1 cells, which are a human, near-haploid, chronic myeloid leukemia cell line (Horizon Discovery, Cambridge, UK).
(A) Typical Western blots from HAP1 cells lacking all IP_3Rs (3KO) or wild-type (WT) cells, using antisera selective for each IP_3R subtype or for β-actin. M_r markers (kDa) are shown. Similar results were obtained in 3 independent analyses.
(B) Ca^{2+} uptake into the ER of saponin-permeabilized cells was recorded in cytosol-like medium, after addition of ATP, using a low-affinity Ca^{2+} indicator (Mag-fluo-4) trapped within the ER lumen (Tovey et al., 2006). The effect of IP_3 (10 μM) on Ca^{2+} release is shown for WT and 3KO cells. Typical results from a single experiment. RFU, relative fluorescence units.
(C) Summary results (mean ± SEM, $n = 4$) show concentration-dependent effects of IP_3 on Ca^{2+} release.
(D) HAP1 cells were treated with bafilomycin A_1 (Baf A_1, 1 μM, 1 hr) in HBS before addition of BAPTA (2.5 mM) to chelate extracellular Ca^{2+} and then thapsigargin (1 μM) to inhibit SERCA. Typical results show mean ± SD from 3 wells in a single experiment.
(E,F) Summary results show peak thapsigargin-evoked increase in $[Ca^{2+}]_c$ in WT (E) and 3KO cells (F) as paired observations (each with 3 determinations) and the mean value (n = 5 (E), 4 (F), line). $^{*}P < 0.05$, paired Student's *t*-test.

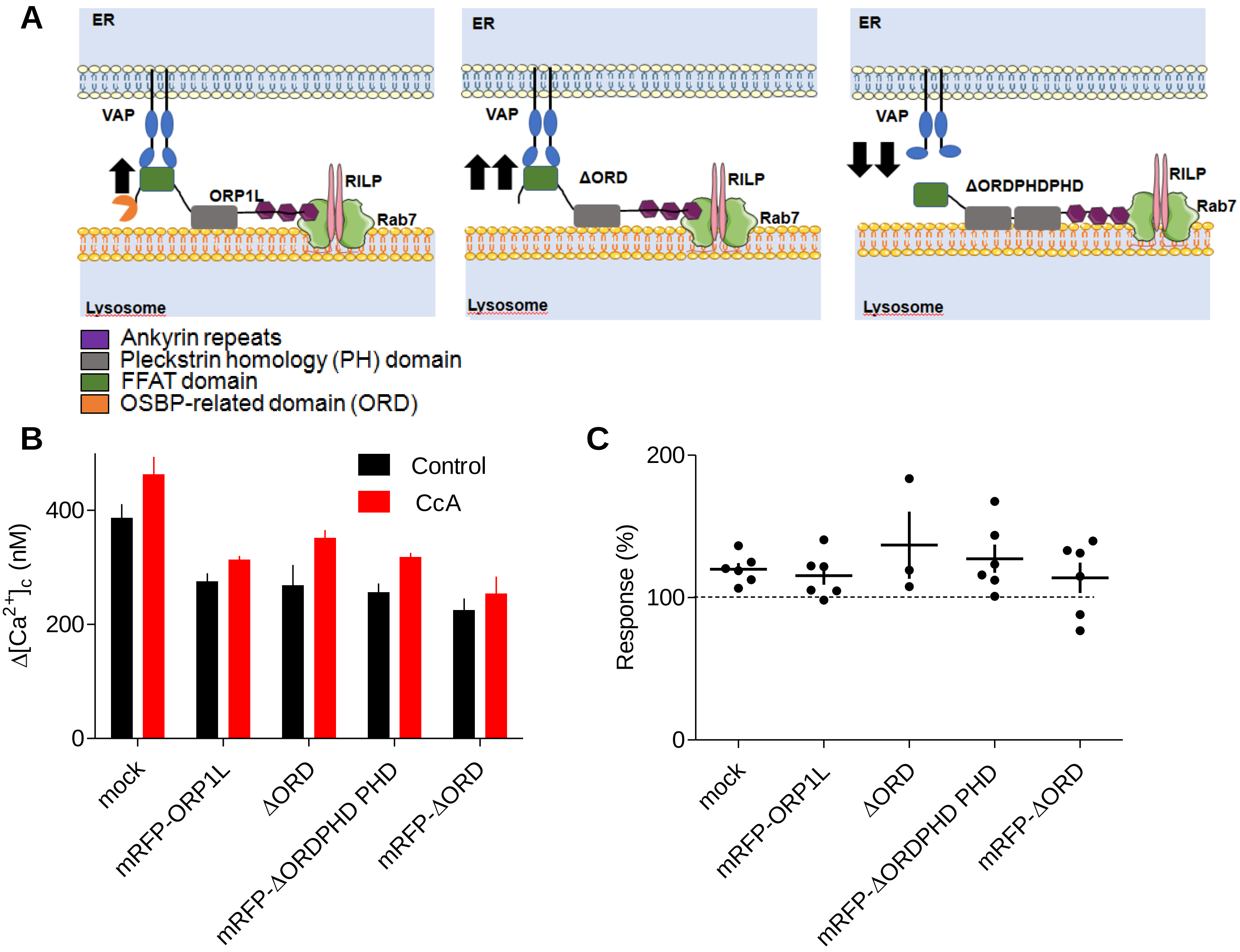

Figure S6. ORP1L is Unlikely to Contribute to ER-Lysosome MCS at which Ca^{2+} Exchange Occurs Related to Figures 4-6

(A) ORP1L associated with rab7 in the lysosome membrane stabilizes ER-lysosome MCS by interacting, through its FFAT domain, with VAP in the ER (Rocha et al., 2009). Cholesterol, by binding to the ORD domain, disrupts the interaction. Hence ORP1L without the ORD domain (ΔORD) forms more stable junctions. The PH domain of ORPL1 binds to the lysosome membrane. Hence, duplicating the domain (ΔORDPHDPHD) destabilizes the MCS.
(B) HEK cells transiently transfected to express ORP1L proteins were stimulated in Ca^{2+}-free HBS with CCh (1 mM) alone or after treatment with CcA (1 µM, 1 hr). Results (mean ± SEM, n = 6 (except for ΔORD, n = 3), each with 3 replicates) show peak increase in $[Ca^{2+}]_c$ evoked by CCh.
(C) For each paired analysis, $\Delta[Ca^{2+}]_c$ evoked by CCh in the presence of CcA was expressed as a percentage of the response to CCh alone (100%).

Our attempts to use siRNA to assess the contributions of ORP1L to ER-lysosome Ca^{2+} exchange were frustrated by the ineffectiveness of two different siRNAs and by the existence of two ORP1 variants. The siRNAs reduced expression of the 55-kDa band by ~17%, but had no significant effect on expression of the 120-kDa band (Johansson et al., 2003).

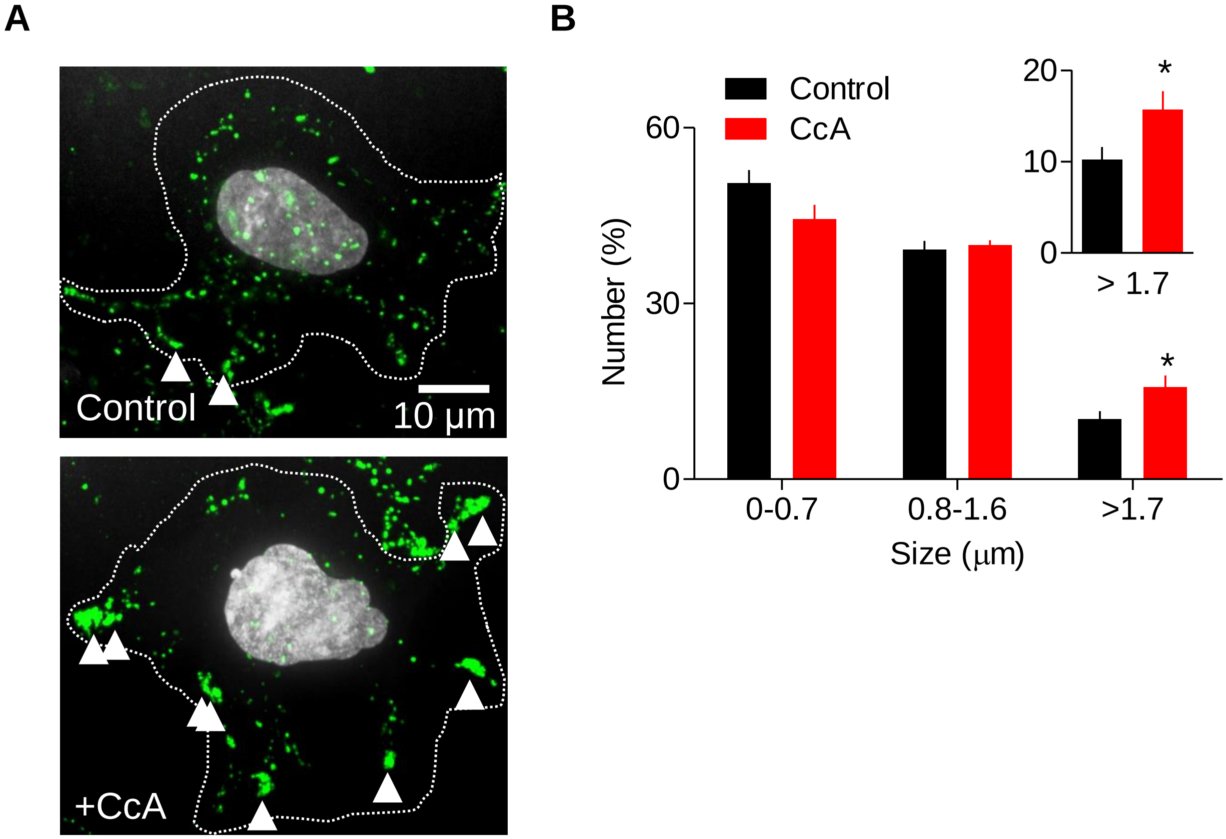

Figure S7. Concanamycin A Causes Redistribution and Enlargement of Lysosomes
Related to Figure 7

(A) Confocal section (close to the coverslip) of HEK cell loaded with Alexa Fluor 488-dextran (10,000 MW) (green) with and without treatment with concanamycin A (CcA, 1 μM, 1 hr). Nuclei, stained with NucBlue are shown in grey. The outline of a single cell is shown. Arrows highlight examples of enlarged lysosomes. Scale bar applies to both images.
(B) Summary results show lysosomes (%) categorized by their Feret diameter (see STAR METHODS). Results are from 653 (control) and 893 (CcA-treated) lysosomes from 7 cells in 3 independent experiments. Inset shows enlargement of the largest size category. $^{*}P < 0.05$, Student's *t*-test, relative to control.
